# Rab25 and CLIC3 Collaborate to Promote Integrin Recycling from Late Endosomes/Lysosomes and Drive Cancer Progression

**DOI:** 10.1016/j.devcel.2011.11.008

**Published:** 2012-01-17

**Authors:** Marta A. Dozynkiewicz, Nigel B. Jamieson, Iain MacPherson, Joan Grindlay, Peter V.E. van den Berghe, Anne von Thun, Jennifer P. Morton, Charlie Gourley, Paul Timpson, Colin Nixon, Colin J. McKay, Ross Carter, David Strachan, Kurt Anderson, Owen J. Sansom, Patrick T. Caswell, Jim C. Norman

**Affiliations:** 1 Beatson Institute for Cancer Research, Garscube Estate, Glasgow G61 1BD, UK; 2 Centre for Oncology and Applied Pharmacology, Division of Cancer Sciences and Molecular Pathology, University of Glasgow, Glasgow G61 1BD, UK; 3 West of Scotland Pancreatic Unit, Glasgow Royal Infirmary, Alexandra Parade, Glasgow G31 2ER, UK; 4 Edinburgh Cancer Research Centre, Institute of Genetics and Molecular Medicine, University of Edinburgh, Edinburgh EH4 2XR, UK; 5 Wellcome Trust Centre for Cell-Matrix Research, Faculty of Life Sciences, University of Manchester, Manchester M13 9PT, UK

## Abstract

Here we show that Rab25 permits the sorting of ligand-occupied, active-conformation α5β1 integrin to late endosomes/lysosomes. Photoactivation and biochemical approaches show that lysosomally targeted integrins are not degraded but are retrogradely transported and recycled to the plasma membrane at the back of invading cells. This requires CLIC3, a protein upregulated in Rab25-expressing cells and tumors, which colocalizes with active α5β1 in late endosomes/lysosomes. CLIC3 is necessary for release of the cell rear during migration on 3D matrices and is required for invasion and maintenance of active Src signaling in organotypic microenvironments. CLIC3 expression predicts lymph node metastasis and poor prognosis in operable cases of pancreatic ductal adenocarcinoma (PDAC). The identification of CLIC3 as a regulator of a recycling pathway and as an independent prognostic indicator in PDAC highlights the importance of active integrin trafficking as a potential drive to cancer progression in vivo.

## Highlights

► Activated integrins move from late endosomes/lysosomes to the plasma membrane ► Spatial segregation of pathways recycling active and inactive integrins ► CLIC3 regulates cell migration and invasion ► CLIC3 dictates whether Rab25 functions as cancer progression suppressor or promoter

## Introduction

Metastasizing cells need to invade the extracellular matrix (ECM) that surrounds tissues and tumors, and must survive and grow within these microenvironments (Sahai, 2005). As they invade, tumor cells form dynamic interactions with the ECM that not only provide traction force for forward motion and ECM remodeling but also promote cell growth and survival (Kumar and Weaver, 2009). Both the motility and the growth and survival of tumor cells are controlled by integrins: transmembrane proteins that interact extracellularly with ECM proteins, such as fibronectin and collagen, and intracellularly with the cytoskeleton and the cell's signaling and vesicular transport machinery (Caswell et al., 2009; Hynes, 2002). Although the ECM protein, fibronectin, is not normally found in adult tissue, it is a very abundant component of the tumor-associated ECM (Zetter, 1993), and the cell's major fibronectin-binding integrin (α5β1) is key to the survival (Lee and Juliano, 2000; O'Brien et al., 1996; Zhang et al., 1995) and migration (Caswell et al., 2007, 2008; Muller et al., 2009) of tumor cells.

α5β1 is continuously endocytosed and then returned, or recycled, to the plasma membrane via Rab11- and Arf6- dependent pathways (Powelka et al., 2004; Roberts et al., 2001; Tayeb et al., 2005). Membrane trafficking pathways mediating α5β1 recycling influence its capacity to promote cancer invasion. Expression of p53 mutants or inhibition of αvβ3 integrin can drive recruitment of the Rab11 effector, Rab-coupling protein (RCP), to the cytotail of β1 integrin, which then enables RCP to associate with the EGF receptor 1 (EGFR1). α5β1 and EGFR1 are then coordinately recycled to the plasma membrane in a way that potentiates EGFR1 signaling to Akt, a kinase that promotes invasion (Caswell et al., 2008; Muller et al., 2009), cell growth, and survival.

Another Rab11 family member, Rab25, is associated with aggressive cancers (Caswell et al., 2007; Cheng et al., 2004), and drives invasion by binding to α5β1 to control its recruitment to the tips of invasive projections (Caswell et al., 2007). There is also evidence that Rab25 can suppress tumor progression. Nam et al. (2010) have shown that loss of Rab25 promotes intestinal neoplasia, and low levels of Rab25 are associated with human colorectal cancer. Rao and coworkers (Cheng et al., 2006, 2010) have found that Rab25 expression is reduced in many breast tumors, and its loss in estrogen-receptor-negative breast cancer cell lines promotes aggression. These discrepancies led us to further investigate the mechanisms by which Rab25 influences invasive behavior in vitro and the progression of cancer in vivo. We show that Rab25 directs conformationally active α5β1 integrin to the lysosome. Lysosomally routed α5β1 is not degraded but is rapidly recycled to the plasma membrane via a pathway requiring the Chloride Intracellular Channel Protein 3 (CLIC3). CLIC3 is required for cell migration and is associated with activated integrin signaling both in ex vivo 3D microenvironments and in human tumors. Moreover, CLIC3 levels dictate metastasis and poor patient survival, thus highlighting the importance of integrin trafficking to cancer progression in vivo.

## Results

### CLIC3 Is Upregulated in a Rab25 and 3D Matrix-Dependent Fashion

**Figure 1:**
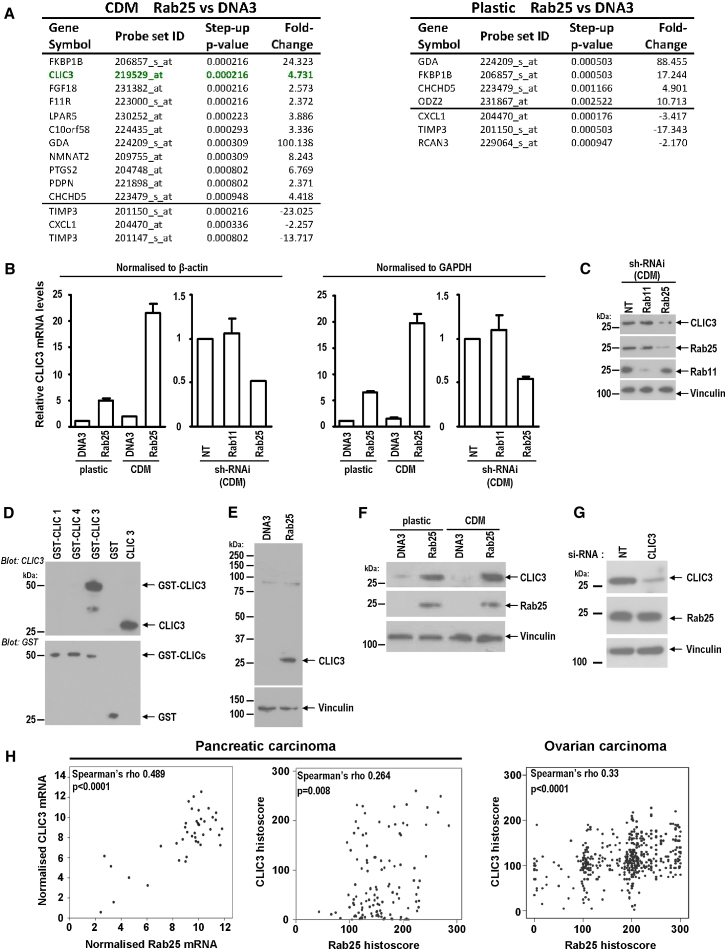
CLIC3 Is Upregulated in Rab25-Expressing A2780 Cells (A) A2780 cells expressing Rab25 or a control vector (DNA3) were seeded onto plastic or CDM, and genes differentially expressed in Rab25 cells were ordered by decreasing p value. Data are mean from three independent experiments. See also Table S1. (B and C) CLIC3 mRNA levels (normalized to β-actin or GAPDH) of cells plated onto plastic or CDM were assessed by qPCR, with Rab11 and Rab25 being suppressed using shRNAi as indicated. Data in (B) are mean ± SEM of three independent experiments. Suppression of Rabs was verified by western blotting. (D) Western blots indicating antibody specificity for CLIC3 (but not GST-tagged CLICs 1 and 4). Protein loading was confirmed by probing with anti-GST. (E) A2780-DNA3 and A2780-Rab25 lysates were immunoblotted for CLIC3 using vinculin as a loading control. (F) Levels of CLIC3 protein in A2780-DNA3 and A2780-Rab25 cells plated onto plastic or CDM were determined by western blotting. (G) Levels of Rab25 protein in A2780-Rab25 cells following CLIC3 suppression (using siRNA) were determined by western blotting. (H) Spearman's correlations indicating a positive relationship between CLIC3 and Rab25 gene and protein expression in patients with PDAC and ovarian carcinoma. Protein expression was assessed by histoscoring, gene expression (mRNA) by microarray hybridization. Spearman's rho and p values are indicated on the plots. See also Figure S1 for validation of in-house antibodies recognizing Rab25 and CLIC3.

We plated A2780 cells stably expressing either Rab25 (A2780-Rab25) or a control vector (A2780-DNA3) onto plastic or cell-derived matrix (CDM)—a thick, pliable matrix composed mainly of fibrillar collagen and fibronectin that recapitulates aspects of the matrix found in connective tissues—and compared their mRNA profiles using Affymetrix arrays. We ranked gene expression changes primarily according to the less conservative step-up p value (one that controls for false discovery rate) and secondarily according to fold changes (see Table S1 available online), and this revealed CLIC3 to be the most significant Rab25-upregulated gene when cells were plated onto CDM, but not following adherence to plastic surfaces (Figure 1A).

To confirm CLIC3 induction, mRNA from A2780-DNA3 and A2780-Rab25 cells was analyzed by real-time quantitative PCR (qPCR). CLIC3 mRNA was induced 6-fold by Rab25 on plastic dishes, and this increased to >20-fold when cells were adherent to CDM (Figure 1B). Moreover, CLIC3 mRNA expression in A2780-Rab25 cells was reduced by siRNA of Rab25 (but not Rab11) (Figures 1B and 1C), indicating that altered regulation of CLIC3 levels was not due to clonal variation between the two A2780 lines but was Rab25 specific.

We generated a highly specific polyclonal antibody that recognized CLIC3, but not CLIC1 and CLIC4: the two CLIC family members most closely related to CLIC3 (Figure 1D). Our anti-CLIC3 recognized a protein of the expected molecular weight (27 kDa) that was abundant in lysates from A2780-Rab25 cells but barely detectable in control cells (Figure 1E). Moreover, CLIC3 protein levels were somewhat increased by plating A2780-Rab25 cells on CDM (Figure 1F), and this was opposed by knockdown of Rab25 (Figure 1C). Conversely, siRNA of CLIC3 did not alter Rab25 levels (Figure 1G).

To evaluate the relationship between Rab25 and CLIC3 expression in tumor cells, we compared their mRNA and protein levels in a large cohort of pancreatic and ovarian tumors (see Figure S1 for validation of Rab25 and CLIC3 antibodies). Rab25 and CLIC3 levels correlated with one another in both pancreatic and ovarian carcinoma (Figure 1H). These data indicate that elevated Rab25 is associated with enhanced CLIC3 expression both in a cell culture model and human tumors.

### Rab25-Dependent Sorting of Ligand-Engaged α5β1 to CLIC3-Positive Late Endosomes/Lysosomes

**Figure 2:**
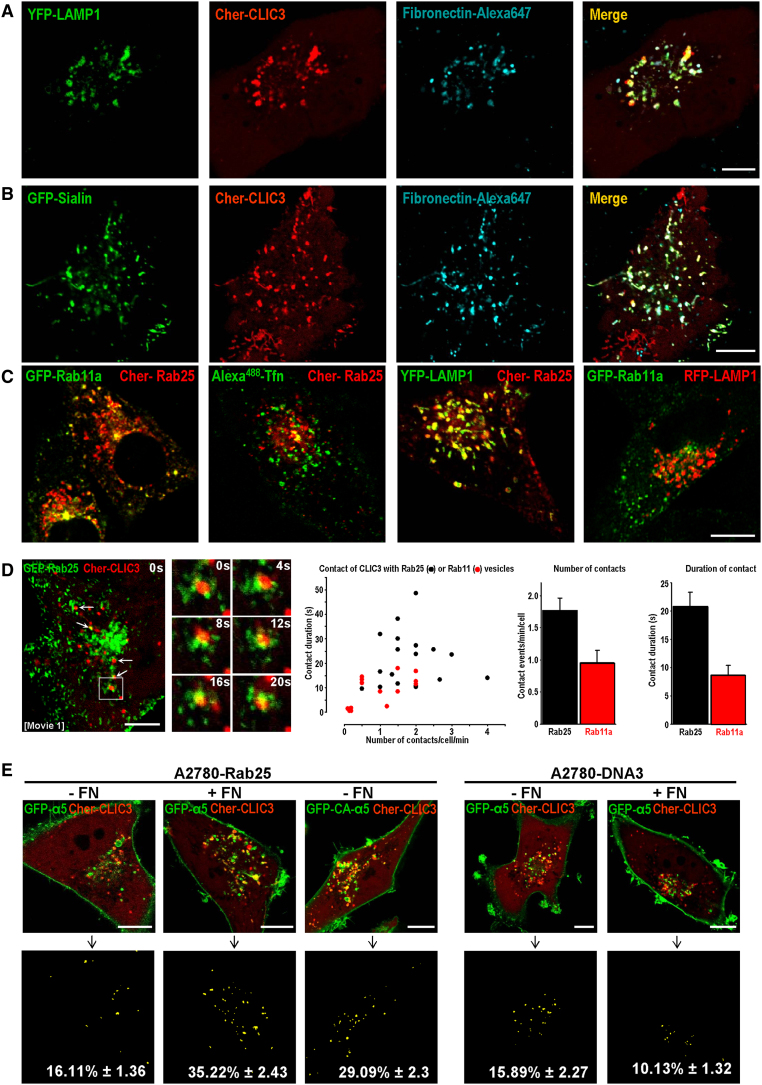
Rab25 Is Required for Sorting of Active α5β1 to CLIC3-Positive Late Endosomes/Lysosomes (A–D) A2780-Rab25 cells were transfected with the indicated fluorescently tagged proteins and incubated with Alexa^647^ fibronectin (2.5 μg/ml) or Alexa^488^ transferrin (10 μg/ml) as indicated. The distribution of the fluorescent proteins was determined by live confocal microscopy. In (A)–(C) single images were acquired, in (D) confocal sections were captured at 2 s intervals over a period of 2 min, and six time points from this sequence (0, 4, 8, 12, 16, and 20 s) are shown. Scale bars, 10μm. The number and duration of contact events between CLIC3-positive structures and Rab25 (black filled circles) or Rab11a (red filled circles) vesicles were quantified and are presented as a scatterplot and a histogram. The stills in (D) are extracted from Movie S1. See also Figure S2. (E) A2780-Rab25 or A2780-DNA3 cells were transfected with Cherry-CLIC3 (red) and either GFP-α5 or GFP-CA-α5 and incubated with or without fibronectin (FN, 2.5 μg/ml). ImageJ was used to quantify colocalization between CLIC3 and α5, as indicated in the lower panel. Colocalization is expressed as a percentage of yellow pixels versus red pixels. Values are mean ± SEM from at least four experiments (n > 30 cells). Scale bars, 10 μm.

In glass-attached A2780-Rab25 cells, Cherry-CLIC3 was not present at early (EEA1) or recycling (Rab11) endosomes (Figures S2A and S2B) but colocalized with the late endosomal marker Rab7 (Figure S2C), and was particularly coincident with LAMP1, Sialin (Figures 2A and 2B), and lysotracker (Figure S2D), which are well-established markers of late endosomes/lysosomes. Many Rab25-positive structures colocalized with Rab11a and internalized transferrin, particularly in the perinuclear region, consistent with previous observations that this GTPase is located at recycling endosomes (Figure 2C) (Casanova et al., 1999). Nevertheless, many Rab25-positive structures did not colocalize with Rab11a or internalized transferrin but displayed marked coincidence with LAMP1 (Figure 2C) and Rab7 (data not shown), indicating that Rab25 was also present at late endosomes. Moreover, Rab11a did not colocalize with LAMP1, indicating that late and recycling endosomes are morphologically distinct in these cells (Figure 2C). Examination of time-lapse movies indicated a relationship between Rab25 and CLIC3-positive endosomes, such that the two structures appear frequently to contact one another and remain in close apposition for ≈20 s at a time (see arrows and inset in Figure 2D; Movie S1). We have quantified the number and duration of contact events occurring between Rab25 and CLIC3 structures, and compared these with those observed between the CLIC3 compartment and Rab11 recycling endosomes. Rab25 endosomes contact CLIC3 structures on average 1.8 ± 0.2 times/min/cell, and these events persist, on average, for 21 ± 2.4 s (Figure 2D). By contrast, contact events between Rab11 endosomes and CLIC3 structures are fewer (0.9 ± 0.2 times/min/cell) and persist for less time (9 ± 2.0 min). Taken together, these data indicate that Rab25 is localized not only to recycling endosomes but also to a late endosomal population that is distinct from Rab11/transferrin endosomes. Furthermore, Rab25-positive structures contact the CLIC3 compartment (which is also late endosomal/lysosomal in nature) significantly more than do recycling endosomes.

Fibronectin, the main ligand for α5β1, is known to be transported to lysosomes following internalization (Shi and Sottile, 2008). Consistently, we found that fluorescently labeled soluble fibronectin accumulated in late endosomes/lysosomes to colocalize closely with CLIC3 (Figures 2A, 2B, and S2C), suggesting that ligand-occupied α5β1 may also be trafficked toward lysosomes. Indeed, quantitative fluorescence imaging indicated that addition of soluble fibronectin to A2780-Rab25 cells promoted delivery of α5β1 to CLIC3-positive late endosomes/lysosomes (Figure 2E). Conversely, in the absence of Rab25, fibronectin did not increase colocalization of α5β1 with CLIC3 (Figure 2E), indicating that Rab25 plays a role in directing fibronectin-occupied α5β1 heterodimers to the lysosomal system.

### The Conformational Status of α5β1 Dictates Its Late Endosomal/Lysosomal Sorting

Binding of fibronectin to integrins leads to structural reorganization such that they assume an active conformation (Moser et al., 2009). Integrin activation can also be induced from within the cell by association of cytoplasmic proteins, such as talin, or by introduction of point mutations that weaken intermolecular interactions between the α- and β-integrin cytotails (Hughes et al., 1996). Previous studies have shown that F1025A mutation in the α5 integrin cytodomain leads to constitutive α5β1 activation (Webb et al., 2007). Using an antibody specific for the active conformation of β1 (9EG7), we found that addition of fibronectin or expression of constitutively active α5 (CAα5) increased the amount of immunoprecipitable active β1 integrin (Figure S3A). This was independent of Rab25 status, indicating that this GTPase does not influence fibronectin's ability to activate β1 integrins.

9EG7 staining colocalized with Sialin in fixed A2780-Rab25 cells (Figure S3B), indicating that active-conformation integrins can be sorted to late endosomes/lysosomes. Consistently, quantitative fluorescence imaging indicated that GFP-tagged CAα5 (GFP-CAα5) integrin was localized to CLIC3-positive late endosomes/lysosomes (Figure 2E) even in the absence of added fibronectin. Moreover, transport of GFP-CAα5 to CLIC3 vesicles was not apparent in A2780 cells that did not express Rab25 (Figure 2E).

These data indicate that active-conformation α5β1 heterodimers are routed to late endosomes/lysosomes where they colocalize with CLIC3, and that Rab25 (which is also located on elements of the late endosomal system) is necessary for this sorting event.

### CLIC3 Controls α5β1 Recycling from Late Endosomes/Lysosomes

**Figure 3:**
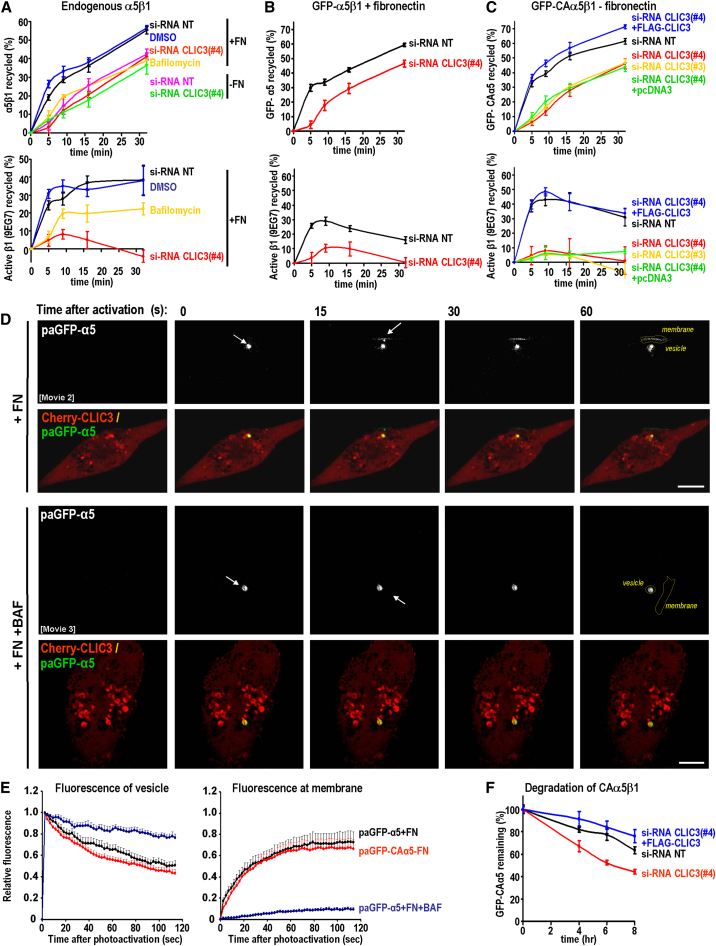
CLIC3 Is Required for Recycling of Activated α5β1 Integrin (A) A2780-Rab25 cells were either left untransfected or were transfected with nontargeting siRNA (siRNA-NT) or with siRNA targeting CLIC3 (siRNA CLIC3#4). They were then surface labeled with 0.2 mg/ml NHS-S-S-Biotin at 4°C, and internalization was then allowed to proceed for 30 min at 37°C in the presence or absence of 3 μg/ml FN, bafilomycin (100 nM), or vehicle control (DMSO). Biotin remaining at the cell surface was removed by exposure to MesNa at 4°C, and the cells were incubated for the indicated times at 37°C. The quantity of biotinylated receptors remaining within the cells was determined by capture ELISA using microtiter wells coated with monoclonal antibodies recognizing α5 or active β1 (9EG7). Values are mean ± SEM (n = 3 independent experiments). (B) A2780-Rab25 cells were transfected with nontargeting (siRNA-NT) or siRNA oligonucleotide targeting CLIC3 (siRNA-CLIC3#4), in conjunction with GFP-α5. Recycling assays were performed in the presence of fibronectin (3.0 μg/ml) as for (A). Biotinylated integrins were detected by capture ELISA using antibodies recognizing GFP or active β1 (9EG7). Values are mean ± SEM (n = 3 independent experiments). (C) A2780-Rab25 cells were transfected with GFP-CA-α5 in conjunction with nontargeting (siRNA-NT) or siRNA oligonucleotides targeting CLIC3 (siRNA-CLIC3#3 and #4), as indicated. CLIC3 suppression with oligonucleotide #4 was rescued using either a control vector (pcDNA3) or siRNA-resistant CLIC3 rescue vector (FLAG-CLIC3). Recycling assays were performed in the presence of fibronectin (3.0 μg/ml) as for (A). Biotinylated integrins were detected by capture ELISA using antibodies recognizing GFP or active β1. Values are mean ± SEM (n = 3 independent experiments). (D and E) A2780-Rab25 cells were transfected with Cherry-CLIC3 in conjunction with photoactivatable α5 integrin (paGFP-α5) or constitutively active α5 integrin (paGFP-CAα5). The cells were incubated with fibronectin (FN; 2.5 μg/ml) or fibronectin in conjunction with bafilomycin (BAF; 100 nM), as indicated. Integrin recycling from CLIC3 vesicles was visualized using photoactivation, which was performed with a 405 nm laser aimed at CLIC3-positive vesicles, as denoted by the white arrows. Images were captured with a confocal microscope every 2 s over a period of 120 s. Movies were generated from these time-lapse images, and stills corresponding to frames prior to photoactivation, immediately after photoactivation (0 s), and subsequently at 15 s intervals are presented. Scale bars, 10 μm (D). The integrated fluorescence intensity of the photoactivated region (vesicle) and adjacent plasma membrane region (membrane) was calculated for each frame of the movie, and these values were plotted against time. Values are mean ± SEM from eight individual experiments (n = 14 for paGFP-α5+FN, n = 43 for paGFP-CAα5, and n = 8 for paGFP-α5 +FN +BAF) (E). See also Movies S2 and S3 and Figure S4A. (F) A2780-Rab25 cells were transfected with GFP-CA-α5 in conjunction with nontargeting (siRNA-NT) or siRNA oligonucleotide targeting CLIC3 (siRNA-CLIC3#4). CLIC3 suppression was rescued using CLIC3 rescue vector (+FLAG-CLIC3), as indicated. Degradation of α5 integrin was monitored over a period of 8 hr using capture ELISA and antibodies recognizing GFP.

When surface-labeled α5β1 was endocytosed in the absence of added fibronectin, it returned to the plasma membrane with largely single order kinetics, and this was unaffected by knockdown of CLIC3 (Figure 3A). However, following inclusion of fibronectin during the internalization period, or expression of CAα5—two situations under which transport of α5β1 to late endosomes/lysosomes is promoted—the integrin recycled with altered kinetics (Figures 3A–3C). There was an initial, rapid component to α5β1's return to the plasma membrane that was reduced by knockdown of CLIC3 using two independent siRNA duplexes, or by addition of bafilomycin, a drug that opposes late endosome/lysosome acidification (Figures 3A–3C). Moreover, the ability of CLIC3 knockdown to oppose recycling of CAα5β1 was negated by expression of an siRNA-resistant “rescue” mutant of CLIC3 (FLAG-CLIC3), but not by a control vector (pcDNA3) (Figure 3C). We then performed trafficking assays using the 9EG7 antibody so as to detect recycling only of active β1 heterodimers. This revealed that active β1 integrins were returned to the plasma membrane with kinetics that corresponded to the rapid, CLIC3-dependent component of recycling (Figures 3A–3C). Moreover, recycling of active integrin was opposed by CLIC3 knockdown (Figures 3A–3C), and this was particularly apparent following expression of CAα5 (Figure 3C). Bafilomycin also suppressed recycling of the 9EG7-positive integrins in a way that was mostly apparent at earlier time points (Figure 3A). These data indicate that CLIC3 is required for recycling of lysosomally targeted α5β1 and that the heterodimers can return to the plasma membrane in their active conformation.

### Visualization of Integrin Recycling from Late Endosomes/Lysosomes in Glass-Attached Cells

To visualize integrin recycling from CLIC3-positive late endosomes/lysosomes in glass-attached A2780 cells, we used photoactivatable GFP-α5 (paGFP-α5). We expressed paGFP-α5 together with Cherry-CLIC3 and incubated the cells with fibronectin to promote transport of the integrin to late endosomes/lysosomes. Alternatively, to visualize recycling of integrins in the active conformation while obviating the need to include fibronectin, we used a photoactivatable GFP version of CAα5 (paGFP-CAα5). We then aimed a 405 nm laser at a “single point” corresponding to a Cherry-CLIC3-positive vesicle, and this led to photoactivation of late endosomally/lysosomally localized wild-type (Figure 3D; Movie S2) or constitutively active (Figure S4A) integrin. Following this, fluorescence was lost from the photoactivated vesicle, which was accompanied by increased fluorescence at an adjacent region of the plasma membrane (Figures 3D and S4A; Movie S2). Quantification of movies indicated that both CAα5 and the fibronectin-engaged wild-type integrin recycled at rates that were indistinguishable (Figure 3E). Consistent with results from biochemical assays, paGFP-α5 remained within the CLIC3 compartment following photoactivation and did not recycle following bafilomycin addition (Figures 3D and 3E; Movie S3).

While addressing the fate of active α5β1 heterodimers following CLIC3 knockdown, we found it impossible to photoactivate integrin in late endosomes/lysosomes following CLIC3 knockdown, suggesting that it had been degraded. To address this, we surface labeled GFP-CAα5 in A2780-Rab25 cells and used a capture ELISA to record its degradation. Active α5β1 was degraded slowly in control knockdown cells, and this was markedly increased following siRNA of CLIC3 (Figure 3F). Moreover, expression of an siRNA-resistant CLIC3 “rescue” construct protected CAα5 from degradation in CLIC3 knockdown cells (Figure 3F).

Taken together, these biochemical and photoactivation-imaging data indicate that “active” conformation α5β1 heterodimers that have been routed to CLIC3-positive late endosomes/lysosomes are not degraded, but rapidly recycled to the plasma membrane, via a mechanism that requires CLIC3 and lysosome acidification. In the absence of CLIC3, lysosomally routed α5β1 heterodimers cannot return to the plasma membrane and are degraded.

### Activated α5β1 Integrins Are Transported Retrogradely during Migration on CDM

A2780-Rab25 cells move on CDM by extending long pseudopods in the direction of migration (Caswell et al., 2007). Fluorescence imaging indicated that Rab25 colocalized closely with LAMP1 (Figure S5A) and Rab7 (data not shown) within the tip and shaft of these pseudopods. Moreover, there were frequent and persistent contact events between Rab25 (but not Rab11) and CLIC3-positive structures that were observable primarily in the pseudopod shaft (see white arrows in Figures S5B and S5C). Furthermore, CLIC3 colocalized with LAMP1 (data not shown) and Sialin (Figure S5D), and these late endosomal/lysosomal compartments moved bidirectionally within the pseudopod shaft, suggesting the possibility that they mediate transport of cargo to or from the cell front.

**Figure 4:**
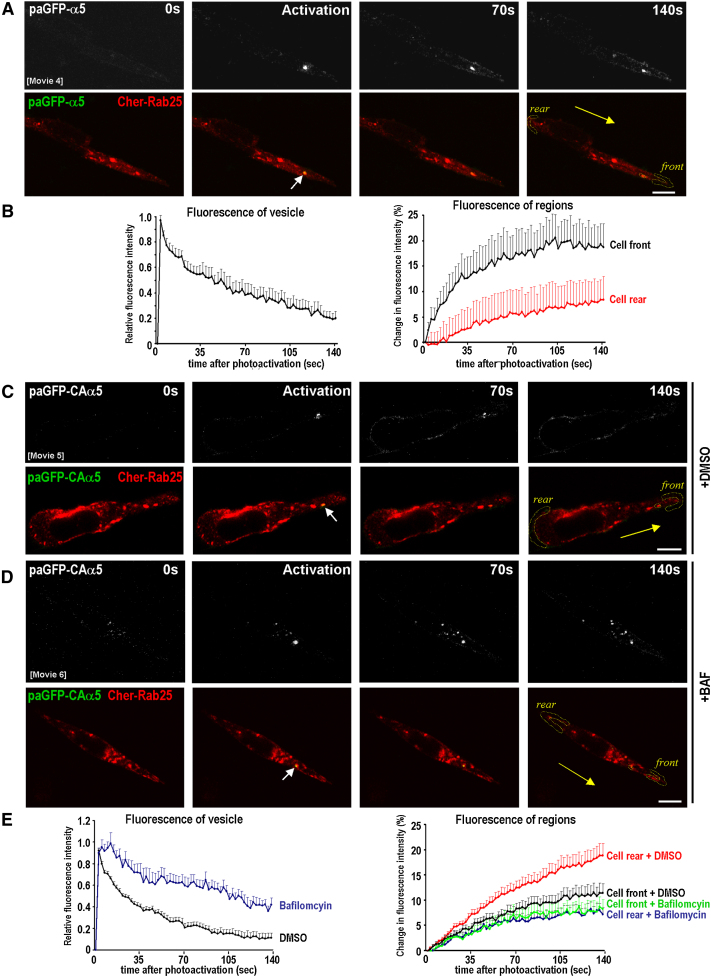
Conformationally Active Integrins Are Trafficked Retrogradely in Cells Migrating on CDMs A2780-Rab25 cells were transfected with Cherry-Rab25 in conjunction with photoactivatable α5 integrin (A; paGFP-wt-α5) or a constitutively active version of the probe (C and D; paGFP-CA-α5). Cells were plated onto CDMs 6 hr prior to imaging and either left untreated (A and C) or treated with bafilomycin (D). Integrin recycling from Rab25 vesicles toward the tips of extending pseudopods was visualized using photoactivation, which was performed with a 405 nm laser aimed at Rab25-positive vesicles, as denoted by the white arrows. Images were captured with a confocal microscope over a period of 140 s. Movies were generated from these time-lapse images, and stills presented correspond to frames prior to photoactivation, immediately after photoactivation, and subsequently at intervals as indicated. Scale bars, 10 μm. Yellow arrow indicates the direction of migration. The integrated fluorescence intensity of the photoactivated region and plasma membrane regions at the cell front and cell rear (yellow dotted lines) was calculated for each frame of the movie. Relative fluorescence intensity (vesicle) or percent increase in fluorescence intensity (region) was plotted against time (B and E). Values are mean ± SEM from more than three individual experiments (n = 6 for paGFP-wt-α5, n = 19 for paGFP-CA-α5 +DMSO, n = 11 for paGFP-CA-α5 +BAF). See also Movies S4, S5, and S6.

We photoactivated paGFP-α5 within Rab25 vesicles near the pseudopod tip and, consistent with previous observations, paGFP-α5 recycled preferentially at the cell front (Figures 4A and 4B; Movie S4). By contrast, paGFP-CAα5 did not recycle primarily at the pseudopod tip but was retrogradely trafficked to the cell rear (Figures 4C and 4E; Movie S5). Retrograde trafficking of paGFP-CAα5 was opposed by bafilomycin (Figures 4D and 4E; Movie S6), which blocks recycling of active α5β1 in glass-attached cells (Figure 3). Indeed, in the presence of bafilomycin, paGFP-CAα5 did not efficiently recycle at the cell rear but appeared to move slowly from the photoactivated vesicle to more rearward late endosomes (Figure 4D) that were LAMP1 and CLIC3 positive (Figure S4B). Further experiments indicated that paGFP-CAα5 photoactivated within Cherry-CLIC3 vesicles toward the cell front did not recycle at the pseudopod tip but was destined for other intracellular compartments and the plasma membrane in the cell rear (Figure S4C).

Taken together, these data indicate that when cells migrate on CDM, wild-type α5β1 is recycled at the cell front, whereas integrins locked in the active conformation are transported backward via the CLIC3/lysosomal pathway and recycled at the rear.

### CLIC3 Is Required for Migration on CDM and for Invasion into 3D Microenvironments

**Figure 5:**
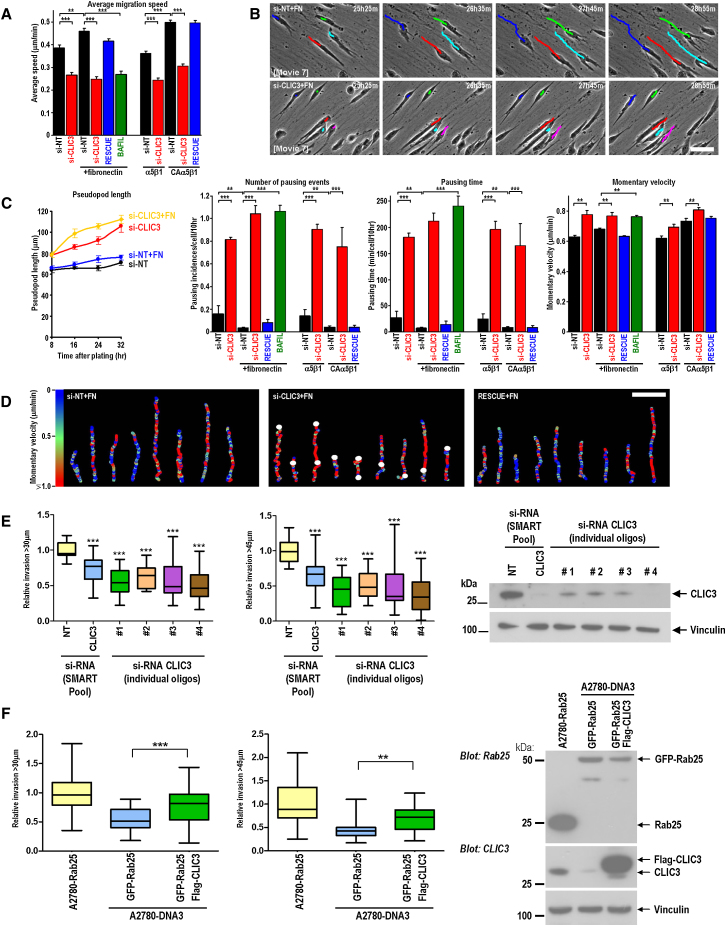
Knockdown of CLIC3 Causes Migrating Cells to Pause and Suppresses Invasion into Fibronectin-Supplemented Matrigel (A–D) A2780-Rab25 cells were transfected with nontargeting siRNA (si-NT) or with an siRNA targeting CLIC3 (oligo#4) (si-CLIC3), in combination with α5 integrin (α5β1), constitutively active α5 integrin (CAα5β1), and an siRNA-resistant CLIC3 rescue vector (rescue) as indicated. Transfected cells were plated onto CDM in the presence or absence of soluble fibronectin (2.5 μg/ml), with or without bafilomycin (10 nM), and allowed to adhere for 24 hr prior to time-lapse microscopy. Images were captured every 5 min over an 8 hr period, and movies were generated from these. Excerpts from these movies are displayed in (B) (Movie S7). Scale bar in (B) is 100 μm. The position of the cell nucleus was followed using cell-tracking software, and the average migration speed (A) and the distance between the center of the nucleus and the cell front (with respect to the direction of migration) (B; pseudopod length) were measured. We used in-house software to identify periods during which cells moved less than 3 μm in 90 min and defined this as a “pause” in cell migration. The number and duration of pausing events are plotted in (C). The speed that cells move when they are not pausing is termed the “momentary velocity,” and these values are also plotted in (C). Values are mean ± SEM (n > 90 track plots); ^∗∗∗∗^p < 0.001, ^∗∗^p < 0.005, Mann-Whitney U test. Representative migration tracks are displayed and aligned (with cell movement running from the bottom to the top); the momentary velocity is denoted by a color code, the scale of which is indicated on the left side of (D). The points at which cells moved less than 3 μm in 90 min (pauses) are indicated by white dots in (D). Scale bar, 200 μm. See also Movie S7. (E and F) A2780-Rab25 cells were transfected with nontargeting (NT), CLIC3 “SMARTpool,” and four individual siRNA oligonucleotides targeting CLIC3 (#1–4) (E). A2780-Rab25 cells were transfected with an empty vector and A2780-DNA3 cells with GFP-Rab25 or GFP-Rab25 and Flag-CLIC3 in combination (F). The invasiveness of transfected cells into fibronectin-supplemented (25 μg/ml) Matrigel was determined using an inverted invasion assay. Invasion is expressed as the proportion of cells that migrate further than 30 and 45 μm. Data are mean ± SEM from at least three individual experiments, each performed in triplicate; ^∗∗∗∗^p < 0.001, ^∗∗^p < 0.005, Mann-Whitney U test. CLIC3 protein levels were assessed by western blotting at the endpoint of the invasion assay (72 hr following transfection).

When CLIC3 knockdown A2780-Rab25 cells were plated onto CDM, they moved, on average, more slowly than control cells, and this was rescued by re-expression of an siRNA-resistant form of CLIC3 (Figure 5A). Moreover, pseudopods at the front of CLIC3 knockdown (but not control) cells became longer with increasing plating time on CDM (Figures 5B and 5C). We noticed that CLIC3 knockdown cells tended to remain stationary for long periods, but following these pauses they moved quickly forward as the cell body was released (Figure 5B; Movie S7). To quantify this, we defined a cell that moved less than 3 μm within 90 min as one that was engaged in “pausing.” CLIC3 knockdown or addition of bafilomycin markedly increased the duration and incidence of these pauses, and this was restored to control levels by re-expression of CLIC3 (Figures 5C and 5D). Furthermore, the limited pausing that was exhibited by control cells was reduced to almost negligible levels by addition of soluble fibronectin or expression of CAα5 integrin (Figure 5C), indicating that CLIC3's ability to transport active integrin contributes to the suppression of cellular pausing.

To determine the effect of CLIC3 knockdown on cell movement during the period in which cells were moving, we calculated the frame-to-frame displacement of cells while they were not pausing, and we have termed this the “momentary velocity.” Consistent with our observations that CLIC3 cells tend to “jump” forward following release of the cell body and rear (Figure 5B; see arrow in Movie S7), we found that momentary velocity was increased following CLIC3 knockdown or bafilomycin addition (Figures 5C and 5D). These data indicate that CLIC3 is required to coordinate cell body translocation with pseudopod extension to enable pause-free cell migration.

A2780-Rab25 cells invade fibronectin-supplemented Matrigel in an α5β1-dependent fashion (Caswell et al., 2007). Suppression of CLIC3 (with four independent siRNA sequences, or a “SMARTPool”) consistently reduced the penetration of A2780-Rab25 cells into fibronectin-supplemented Matrigel over a 48 hr period (Figure 5E). Transient expression of GFP-Rab25 (which is insufficient to drive CLIC3 expression) had limited capacity to invade fibronectin-supplemented Matrigel (Figure 5F) (Caswell et al., 2007). We therefore tested whether CLIC3 could collaborate with Rab25 to drive invasion when expressed transiently. Indeed, A2780 cells transiently expressing both GFP-Rab25 and FLAG-CLIC3 displayed similar invasiveness to the stable A2780-Rab25 (endogenously CLIC3-expressing) cells (Figure 5F).

**Figure 6:**
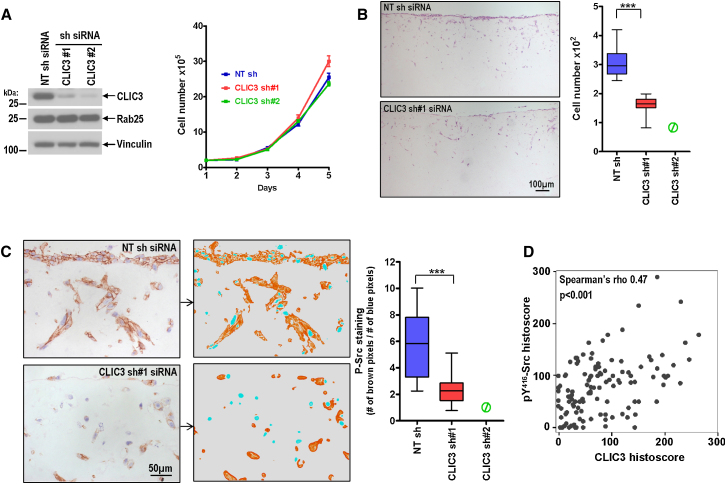
CLIC3 Is Required to Maintain Src Signaling and for Growth and Survival of Cells in Organotypic Matrices (A–C) The effect of CLIC3 suppression on growth and survival of A2780-Rab25 cells in rat tail collagen preconditioned with human primary fibroblasts was assessed using stable knockdown cell lines (A). A2780-Rab25 cells were stably transfected with nontargeting shRNAi (NTsh) or a shRNA vector targeting CLIC3 (CLIC3 sh#1 or sh#2). Protein levels were determined by western blotting, and growth rates on plastic surfaces were assessed by cell counting. Cells were allowed to invade into the collagen matrix for 14 days, and invasion was assessed by counting the number of cells remaining in the plug (B). Phospho-Src staining of NTsh and CLIC3sh clones grown in the collagen matrix and quantification of phospho-Src positivity (C). Representative images and intermediate composite projections that were used for quantification with ImageJ software are shown. Nuclear staining served as internal control, and phospho-Src positivity was expressed as a ratio of brown pixels (phospho-Src) versus blue pixels (hematoxylin nuclear staining). (D) Spearman's correlation indicating relationship between CLIC3 levels and membranous phosphotyrosine^416^-Src in pancreatic adenocarcinoma.

To determine whether CLIC3 influences the ability of tumor cells to thrive in 3D microenvironments over a more prolonged period, we generated A2780-Rab25 lines that stably express small hairpin RNA (shRNA) duplexes to suppress CLIC3 levels (Figure 6A). These CLIC3 knockdown lines had similar capacity to grow and survive on plastic surfaces as control knockdown cells (Figure 6A). We then assessed growth and invasiveness of these cells in organotypic gels formed from acid-extracted rat tail collagen preconditioned with human dermal fibroblasts that are thought to closely recapitulate a tumor stromal environment (Marsh et al., 2008). Control A2780 cells moved into organotypic matrices and survived in considerable numbers over a 14 day period (Figure 6B). By contrast, CLIC3 knockdown clone #1 cells invaded and survived in reduced numbers, and knockdown clone #2 cells (which displayed a much greater reduction of CLIC3 protein) were undetectable following a 14 day incubation period (Figure 6B). These data indicate that CLIC3 is required for movement of Rab25-expressing A2780 cells into Matrigel, and also for their sustained growth and survival within organotypic fibroblast-conditioned 3D matrices.

Activated integrins can alter cell-invasive behavior by recruiting focal adhesion kinase leading to activation and autophosphorylation of Src on tyrosine^416^. Integrin-mediated Src activation is required for tumor cell invasion (Brunton and Frame, 2008), and the use of phospho-specific antibodies to detect levels of phosphotyrosine^416^-Src is a robust way to determine integrin signaling in tissue sections. Active phospho-Src was clearly visible at the plasma membrane of A2780-Rab25 cells both within and near the top of the organotypic plug (Figure 6C). However, in CLIC3 knockdown cells levels of phospho-Src were significantly reduced, as indicated by quantitative analysis of anti-phospho-Src immunohistochemical signal (brown pixels) with respect to the hematoxylin stain (blue pixels) (Figure 6C). Importantly, the histoscore values for CLIC3 and phospho-Src were strongly correlated with one another in pancreatic adenocarcinoma (Figure 6D), indicating the possibility that CLIC3 is associated with upregulated integrin signaling in vivo.

### CLIC3 Expression in Ovarian and Pancreatic Cancer

**Figure 7:**
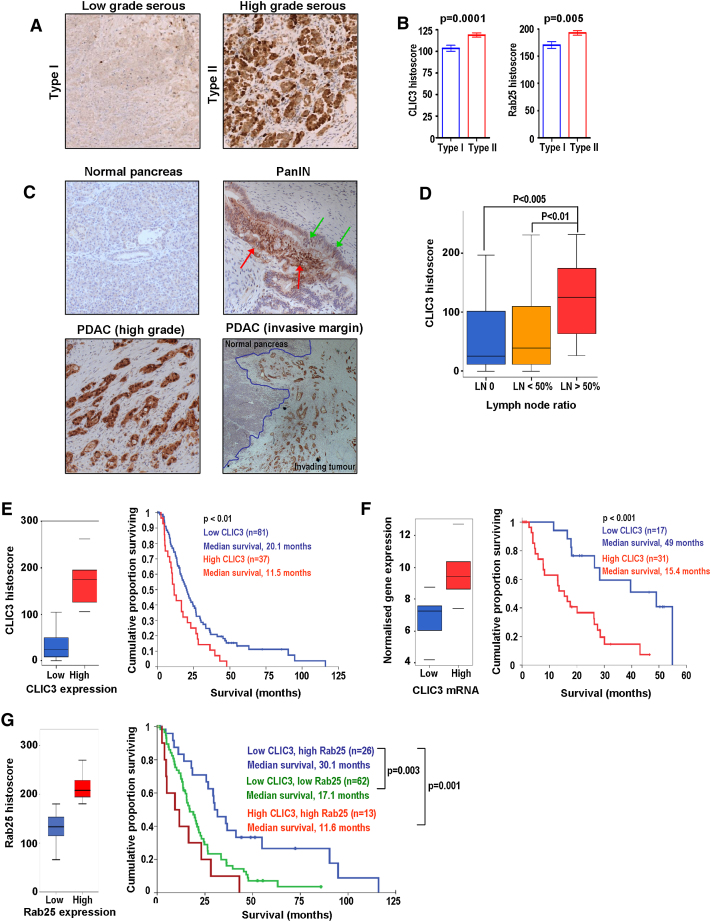
CLIC3 Expression Predicts Poor Patient Survival (A and B) CLIC3 immunohistochemistry of low-grade (type I) and high-grade (type II) serous ovarian cancers. The graphs illustrate the difference in CLIC3 and Rab25 histoscores between type I and type II ovarian tumors (Mann-Whitney U test, p < 0.001 and p = 0.005, respectively). (C) CLIC3 levels in normal pancreatic tissue, PanIN, and PDAC. Green arrows indicate preserved normal structure of ductal epithelium; red arrows point to dysplastic areas. H&E and CLIC3 staining of an invasive margin of a high-grade PDAC. (D) Box plot indicating that tumors from patients with more than 50% lymph node involvement have increased levels of CLIC3. (E) Box plot illustrating stratification of patients with PDAC into low and high CLIC3 expressors based on histoscore. Kaplan-Meier analysis indicates that patients with high CLIC3 expression (n = 37) have a poorer outcome than those with low expression (n = 81) following tumor resection (p < 0.01). (F) Box plot illustrating stratification of patients with PDAC into low and high CLIC3 expressors based on normalized mean gene expression. Kaplan-Meier analysis showing that patients with high CLIC3 mRNA expression (n = 31) have a poorer survival than those with low expression (n = 17) following tumor resection (p = 0.001). (G) Box plot illustrating stratification of patients with PDAC into low and high Rab25 expressors based on histoscore. Kaplan-Meier analysis of low CLIC3 and high Rab25 expressors (blue), low CLIC3 and low Rab25 expressors (green), and high CLIC3 and high Rab25 expressors (red).

Because we have found that CLIC3 acts to recycle integrins that have been targeted to late endosomes/lysosomes by the action of Rab25, we wished to evaluate the expression of CLIC3 in cancers thought to be driven by Rab25. High levels of Rab25 have been shown to correlate with poor prognosis and aggressiveness of ovarian cancers (Cheng et al., 2004), so we initially looked at CLIC3 in patient samples from this disease. We detected CLIC3 expression in various types of ovarian tumors including serous (Figure 7A), endometrioid, clear cell, and mucinous carcinoma (data not shown) primarily in punctate cytoplasmic structures but also within the nucleus of some tumors. Ovarian tumors can be categorized into type I, which includes low-grade micropapillary serous carcinoma, mucinous, endometrioid, and clear cell carcinomas, and type II, which comprises high-grade serous carcinomas, malignant mixed mesodermal tumors, and undifferentiated carcinomas and is commonly highly invasive and much more likely to present with disseminated disease (Vang et al., 2009). Histoscore analysis indicated that CLIC3 protein levels were higher in type II than in type I ovarian tumors (Figure 7B), supporting a potential role for this protein in cancer invasion. Moreover, Rab25 levels were similarly elevated in type II tumors, further supporting the close relationship between this GTPase and CLIC3 (Figure 7B).

Given the histological and pathophysiological similarities between ovarian and pancreatic cancers (Hruban et al., 2007), we analyzed CLIC3 expression in neoplastic lesions of the human pancreas. CLIC3 was undetectable in ductal and acinar tissues of normal human pancreas (Figure 7C). Pancreatic cancers are thought to arise via precursor lesions called pancreatic intraepithelial neoplasms (PanINs) (Hruban et al., 2000). Examination of early PanINs revealed that CLIC3 expression was low within well-organized epithelia (Figure 7C, green arrows), whereas dysplastic PanIN regions were more abundant in CLIC3 (Figure 7C; red arrows). CLIC3 was highly expressed in pancreatic ductal adenocarcinoma (PDAC) (Figure 7C), being localized to cytoplasmic granules but also observable in the nucleus of some cancers with high CLIC3 levels. Moreover, CLIC3 expression was highly enriched in regions where the tumors were invading normal tissue (Figure 7C), suggesting a role for CLIC3 in the invasive/metastatic behavior of pancreatic cancer.

### CLIC3 Predicts Metastasis and Poor Survival in PDAC

To determine the relationship between CLIC3 levels and metastasis in vivo, we performed immunohistochemistry on a tissue microarray (TMA) containing 118 cases (6 cores per patient) of PDAC. Levels of CLIC3 did not differ in terms of tumor size, grade, stage, vascular invasion, or resection margin status. In univariate analysis, high CLIC3 expression at both the protein (Figure 7E) and mRNA (Figure 7F) levels was associated with significantly decreased survival following resection of PDAC. Moreover, tumors removed from patients presenting with a lymph node ratio >50% had elevated levels of CLIC3 by comparison with those with lower levels of lymph node involvement (Figure 7D). Most importantly, in multivariate Cox proportional-hazards regression analysis, high CLIC3 expression remained an independent predictor of poor survival (Table S2).

Rab25 has been reported to both promote and oppose tumor progression. We considered whether these conflicts in the literature may, in part, be explained by Rab25's capacity to elevate CLIC3 levels in the tumor in question. Indeed, although the correlation between Rab25 and CLIC3 in pancreatic tumors is highly statistically significant, this relationship is not hard and fast, and there are many tumors that express Rab25 that do not express CLIC3 and vice versa (Figure 1H). In low CLIC3 expressors, high Rab25 levels were associated with significantly increased patient survival (Figure 7G). Conversely, Rab25 levels do not predict good clinical outcomes when CLIC3 is expressed (Figure 7G). Taken together, these data indicate that in the absence of CLIC3, Rab25 acts as a tumor suppressor. However, by driving CLIC3 expression, Rab25 increases tumor aggressiveness.

## Discussion

Here we demonstrate a pathway for trafficking of activated α5β1 integrins in tumor cells that relies first on the ability of Rab25 to sort active heterodimers toward lysosomes, and then on CLIC3 to mediate the return of α5β1 from late endosomes/lysosomes to the cell surface. Routing of receptors to lysosomes normally leads to their degradation. Indeed, a recent publication has shown that activated α5β1 integrins are ubiquitinylated, trafficked to lysosomes, and degraded (Lobert et al., 2010). We concur that this is indeed the fate of lysosomally targeted integrins in the absence of CLIC3 expression. However, Rab25 is associated with elevated CLIC3 in both cultured A2780 cells and in pancreatic and ovarian tumors, and CLIC3 functions to return lysosomally targeted α5β1 to the plasma membrane, thus opposing receptor degradation. Moreover, although it appears that fibronectin and α5β1 part company during passage through the late endosome/lysosome (data not shown), photoactivation experiments and biochemical recycling assays indicate that the integrin need not, and does not, return to the inactive conformation prior to recycling. Thus, rather than switching conformations as they move through the endosomal pathway, integrins may be segregated into spatially distinct cycling pools according to their activation state, with inactive integrins moving toward the cell front and active ones being transported retrogradely by the late endosomal system.

It is reasonable to propose that α5β1 that is recycled at the front contributes to pseudopod elongation, presumably by engaging fresh ECM. However, effective cell migration needs coordination of pseudopod extension with release/retraction of the cell rear. Following CLIC3 knockdown, although advancing pseudopods continue to extend, the cell body does not efficiently translocate, causing migrating cells to pause for extended periods. These data are consistent with a role for retrograde integrin transport in retraction of the cell rear and/or release of the cell body from the substratum. In addition to promoting cell attachment, activated integrins are responsible for signaling events, such as Src activation, which lead to disassembly of adhesions, and CLIC3 is required to maintain active Src in invading cells. Thus, it is possible that CLIC3-dependent retrograde integrin transport leads to activation of kinases such as Src at the cell rear to allow adhesion disassembly. It may seem counterintuitive to propose that delivery of an activated adhesion receptor would act to reduce, rather than increase, adhesion. However, addition of plasma fibronectin markedly decreases the pausing of cells migrating on CDM, and this may be owing to the ability of this soluble ligand to increase the quantity of active α5β1 that is engaged in endocytic trafficking and signaling to the adhesion disassembly machinery while decreasing that available for attachment to the substratum. By comparison with other integrins, α5β1 is particularly effective at binding to soluble versus immobilized ligand (Huveneers et al., 2008), and this property may thus contribute to α5β1's capacity to promote invasion.

CLIC3's role in Rab25-driven invasiveness and growth/survival in organotypic matrices in vitro suggests that recycling of lysosomally routed α5β1 contributes to metastasis in vivo. This is further supported by our observations that CLIC3 is enriched at tumor invasive margins, and that its expression correlates with lymph node metastases and subsequent death from disseminated disease. Signaling downstream of activated integrins cannot only promote cell migration but also other processes that are associated with metastasis, such as cell growth and survival. For instance, Src promotes cell transformation by activating the transcription factor, STAT3, which, in turn, leads to enhanced expression of genes such as cyclin D1 (Sinibaldi et al., 2000). It is likely, therefore, that increased cycling through the CLIC3 pathway may contribute not only to cell migration but also to growth and survival of metastases by enhancing the capacity of active α5β1 to activate Src and its downstream effectors. Furthermore, it is now apparent that α5β1-dependent Src activation contributes to suppression of apoptosis in suspended cells (Haenssen et al., 2010), further supporting a role for a population of mobile “trafficking” heterodimers in activating signaling in a way that does not require engagement with a substratum.

There are conflicts in the literature as to whether Rab25 functions primarily as a promoter or suppressor of cancer. Loss of Rab25 is associated with tumor initiation in the colon (Nam et al., 2010) and ER-negative breast cancer (Cheng et al., 2006, 2010). Conversely, Rab25 expression correlates with decreased survival and increased aggressiveness of ovarian cancer (Cheng et al., 2004), and enhanced invasive migration of ovarian cancer cells in vitro (Caswell et al., 2007). The present study may resolve some of these conflicts. We propose that in tumors where CLIC3 is absent, Rab25 will act to route active integrins to lysosomes whereupon they will be degraded, thus terminating integrin signaling. Under these circumstances Rab25 will likely act as a tumor suppressor. Conversely, in tumors where Rab25 drives upregulation of CLIC3, lysosomally routed active integrins will be returned to the plasma membrane, thus avoiding degradation and enabling their continued signaling to drive tumor progression. Further work will be necessary to define other key molecular components of the CLIC3-regulated recycling pathway, and to determine how these can dictate Rab25's capacity to promote or retard cancer progression.

## Experimental Procedures

### Cell Culture and Transfection

Stable clones of A2780-DNA3 and A2780-Rab25 cells were generated as described previously. A2780 cells were cultivated in RPMI supplemented with 10% (v/v) serum at 37°C and 10% CO_2_. Transfection of all vectors, small hairpin RNA interference (shRNAi), and siRNA oligonucleotides (Dharmacon) was carried out using the Amaxa Nucleofector system (solution T, program A-23) according to manufacturer's instructions. CDMs were generated as described previously (Bass et al., 2007; Cukierman et al., 2001), but in many experiments telomerase-immortalized fibroblasts were substituted for primary cultured human dermal fibroblasts without any noticeable alteration to the results.

### qPCR Analysis

Cells were plated onto plastic and CDMs for 16 hr, and RNA was extracted using RNeasy kit (QIAGEN). cDNA was synthesized using ImpromII kit (Promega). qPCR was performed using SYBR green (QIAGEN), QuantiTect Primer Assay kits (QIAGEN), and a Chromo4 Engine (Bio-Rad). ΔΔC(t) method was used to calculate changes in gene expression with β-actin and GAPDH serving as reference genes.

### Antibody Generation, Immunohistochemistry, and Histoscore Analysis

Recombinant GST-CLIC3 and His-tagged Rab25 were expressed in BL21(DE3)pLysS *E*. *coli* (GST-tag was removed with PreScission Protease), and the Rab25 and CLIC3 proteins were affinity purified and used for rabbit immunization (Eurogentec). Formalin-fixed and paraffin-embedded tissue sections were dewaxed in xylene and passed through ethanol (2× 100%, 1× 70%) for rehydration. Heat-induced epitope retrieval was carried out in TRIS-EDTA (pH 8) buffer. Endogenous peroxidase was then blocked using 3% H_2_O_2_/methanol for 5 min before polyclonal rabbit CLIC3-specific antibody was applied at 1:500 dilution for 45 min at room temperature. Tissue sections were then incubated in secondary antibody (Dako Envision rabbit kit, K4003) for 40 min, and the staining was visualized with DAB and counterstained with Gills Hematoxylin.

### TMA

The human pancreaticobiliary and ovarian TMAs were produced in the West of Scotland Pancreatic Unit, Glasgow Royal Infirmary, and the Experimental Cancer Medicine Centre, Western General Hospital Edinburgh, respectively. The respective local Research Ethics Committees approved tissue sample collection. A total of 1,500 cores from 224 patients with pancreaticobiliary cancer (including 118 cases with PDAC) were arrayed on the TMA. From each patient at least six tumor cores (0.6 mm diameter) and two cores of adjacent normal tissue were sampled. For the ovarian TMA, 455 single cores were taken from representative tumor areas identified by a specialist gynecological pathologist and arrayed on two blocks. Full clinicopathological parameters and complete follow-up data were available for both tissue sets. To compare the length of survival between the two pancreatic expressor groups, a log rank analysis was performed. To adjust for competing risk factors, a Cox proportional hazards model and univariate analysis were used, and hazard ratios (HRs) with 95% confidence interval (CI) were reported as an estimate of the risk for disease-specific death. Clinicopathological parameters that were identified as significant (p < 0.10) in univariate analysis were incorporated into the multivariate Cox regression analysis in a backward stepwise fashion. Statistical significance for independent outcome predictors was set at p ≤ 0.05. Statistical analyses were performed with the SPSS version 15.0 package (SPSS, Chicago, IL, USA).

For the quantification of CLIC3 and Rab25 RNA levels in pancreatic tumors, fresh tumor tissue was collected from a more recent cohort of 48 patients with PDAC following pancreaticoduodenectomy along with 10 normal pancreatic specimens. Cancerous and normal tissue was frozen and stored at the Glasgow Royal Infirmary Biobank storage facility and subsequently underwent RNA extraction and microarray analysis (Whole Human oligo-Microarray Kit 4 × 44K multiplex format; Agilent Technologies, Santa Clara, CA, USA). A comparison between normal tissue and PDAC specimens revealed that CLIC3 was overexpressed in the PDAC specimens. An unsupervised Kaplan-Meier survival analysis identified CLIC3 as being associated with outcome. Using the median CLIC3 mRNA expression value of the PDAC specimens as a cutoff, the cohort stratified into two groups with significantly different survival outcomes.

### Cell Imaging and Photoactivation

A2780 cells were seeded onto glass-bottomed 3 cm plates and imaged with a 64× objective of an inverted confocal microscope (Fluoview FV1000; Olympus) in an atmosphere of 5% CO_2_ at 37°C. Photoactivation of paGFP-α5 integrin was achieved using a 405 nm laser of that microscope and Olympus SIM scanner. Colocalization quantification was performed using ImageJ software, where the confocal images underwent two rounds of local contrast enhancement (image blurring, subtraction of the blurred image, and subsequent contrast enhancement) and threshold adjustment. The number of yellow pixels was then expressed as a percentage of pixels in the red channel.

### Recycling Assays

Integrin recycling assays were performed as described previously in Roberts et al. (2001). Briefly, A2780-Rab25 cells were serum starved for 1 hr, transferred to ice, washed twice in cold PBS, and surface labeled at 4°C with 0.13 mg/ml NHS-SS-biotin (Pierce) in PBS for 30 min. Following surface labeling, cells were transferred to RPMI containing 10% FCS with or without 2.5 μg/ml bovine fibronectin at 37°C to allow internalization of tracer. Cells were returned to ice and washed twice with ice-cold PBS, and biotin was removed from proteins remaining at the cell surface by reduction with MesNa. The internalized fraction was then chased from the cells by returning them to 37°C RPMI containing 10% FCS with or without 2.5 μg/ml bovine fibronectin at 37°C. At the indicated times, cells were returned to ice, and biotin was removed from recycled proteins by a second reduction with MesNa. Biotinylated integrins were then determined by capture ELISA as follows. MaxiSorp 96-well plates (Life Technologies) were coated overnight with 5 μg/ml of the appropriate anti-integrin antibodies (PharMingen #555651 for α5 integrin or PharMingen 9EG7 for active β1) in 0.05 M Na_2_CO_3_ (pH 9.6) at 4°C and blocked in PBS containing 0.1% Tween 20 (PBS-T) with 5% BSA for 1 hr at room temperature. Integrins were captured by overnight incubation of 50 μl of cell lysate at 4°C. Unbound material was removed by extensive washing with PBS-T, and wells were incubated with streptavidin-conjugated horseradish peroxidase (Amersham) in PBS-T containing 1% BSA for 1 hr at 4°C. Following further washing, biotinylated integrins were detected by chromogenic reaction with ortho-phenylenediamine.

### Inverse Invasion Assay

Inverse invasion assays were performed as described previously (Hennigan et al., 1994). Briefly, complete Matrigel was diluted in an equal volume of ice-cold PBS supplemented with soluble fibronectin (25 μg/ml final concentration). A total of 100 μl of the diluted Matrigel mix was pipetted into a Transwell (8 μm diameter pores), inserted into a well of a 24-well tissue culture plate, and left to set at 37°C. The Transwells were then inverted, and 4 × 10^4^ cells were placed on the underside of the filter. The Transwells were then covered with the base of the 24-well tissue culture plate so that they made contact with cell suspension droplets. Cell attachment was allowed to proceed for 4 hr, before the plate was inverted back and the nonadherent cells were washed off by three sequential washes in 1 ml of serum-free medium. The Transwells were placed in 1 ml of serum-free medium, which constituted the lower chamber of the assay, and 100 μl of 10% FCS-RPMI supplemented with 25 ng/ml EGF was pipetted on top of the Transwell. The cells were then allowed to invade into the Matrigel and toward the gradient of serum and EGF for 48 hr at 37°C in the atmosphere of 5% CO_2_. To visualize cells that migrated into the Matrigel plug, 4 μM Calcein AM (acetoxymethyl ester of calcein) was used. After 1 hr at 37°C, the cells were imaged by confocal microscopy using a Leica SP2 confocal microscope and a 20× objective at an excitation wavelength of 488 nm and emission wavelength of 515 nm. Optical sections were captured at 15 μm intervals, starting from the underside of the Transwell filter and moving upward in the direction of cell invasion. The resulting images were quantified and assembled into invasive strips using ImageJ software. The threshold fluorescence intensity of the images was set to only register cells that lay within each individual optical slice, and the sum of the fluorescence in the sections from 30 or 45 μm and above was divided by the total fluorescence of all the sections, thus giving an invasion index “beyond 30 or 45 μm,” respectively. Data were generated from at least three individual experiments, in which each condition was represented by three Transwells, and optical sections were taken from at least three areas of each Transwell.

### Organotypic Invasion Assays

Approximately 7.5 × 10^4^/ml primary human fibroblasts were embedded in a matrix of rat tail collagen I. Rat tail tendon collagen was prepared by extraction with 0.5 M acetic acid, to a concentration of ∼2 mg/ml. Polymerized matrix was allowed to contract for approximately 7 days and until the fibroblasts contracted the matrix to a plug of approximately 1.5 cm in diameter. Subsequently, 4 × 10^4^ A2780-Rab25 cells were plated onto the matrix and allowed to grow to confluence for 4 days. The matrix was then transferred onto a metal grid and raised to the air/liquid interface. The matrix was thus fed from underneath with complete media supplemented with 25 ng/ml EGF, which was changed every 2 days. After 14 days, the plugs were fixed in 4% PFA, paraffin embedded, sectioned, and stained for H&E or for P-Src and counterstained with hematoxylin.

### Expression Constructs

α5 integrin constructs were expressed as GFP-fusion proteins and were tagged with GFP at the C terminus (cytoplasmic domain). The GFP α5 integrin was generously donated by Donna Webb and is exactly as described in Laukaitis et al. (2001) and Webb et al. (2007). The human gene sequence for CLIC3 (a generous gift from Mark Berryman) was inserted into the pEGFP-C1 vector using XhoI (5′) and BamHI (3′). The EGFP sequence was then replaced with the red fluorescent protein, mCherry, sequence using AgeI (5′) and XhoI (3′) such that the fluorescent tag is attached in frame to the N terminus of CLIC3. The human sequence of CLIC3 was tagged with the FLAG epitope at the N terminus using PCR and then inserted into the pcDNA3 expression vector using BamHI (5′) and Nod (3′). For expression of GST-tagged proteins, the human sequence of CLIC3 was ligated into the pGEX-6P-1 vector using BamHI (3′ to the prescission site and 5′ of the CLIC3 sequence) and Nod (3′ to CLIC3).

## Supplemental Information

Document S1. Figures S1–S5 and Table S2

Table S1. Microarray Sample Preparation, Data Analysis and Validation, and the Full Microarray Data Set, Related to Figure 1

Movie S1, Related to Figure 2. Dynamics of GFP-Rab25 and Cherry-CLIC3 in Glass-Attached A2780 Cells A2780-Rab25 cells were transfected with GFP-Rab25 in combination with Cherry-CLIC3 and plated onto glass dishes. The distribution of the fluorescent proteins was determined by live confocal microscopy with images being captured at 2 s intervals over a period of 2 min. The stills in Figure 2D are extracted from this movie.

Movie S2, Related to Figure 3. Dynamics of Photoactivated paGFP-α5 Integrin in Glass-Attached A2780 Cells A2780-Rab25 cells were transfected with Cherry-CLIC3 in conjunction with photoactivatable α5 integrin (paGFP-α5). Cells were incubated with fibronectin (FN; 2.5 μg/ml). Integrin recycling from CLIC3 vesicles was visualized using photoactivation, which was performed with a 405 nm laser aimed at CLIC3-positive vesicles. Images were captured with a confocal microscope every 2 s over a period of 120 s. Stills corresponding to frames prior to photoactivation, immediately after photoactivation (0 s), and subsequently at 15 s intervals from this movie are presented in Figure 3D.

Movie S3, Related to Figure 3. Dynamics of Photoactivated paGFP-α5 Integrin in Glass-Attached A2780 Cells in the Presence of Bafilomycin A2780-Rab25 cells were transfected with Cherry-CLIC3 in conjunction with photoactivatable α5 integrin (paGFP-α5). Cells were incubated with fibronectin (FN; 2.5 μg/ml) in conjunction with bafilomycin (BAF; 100 nM). Integrin recycling from CLIC3 vesicles was visualized using photoactivation, which was performed with a 405 nm laser aimed at CLIC3-positive vesicles. Images were captured with a confocal microscope every 2 s over a period of 120 s. Stills corresponding to frames prior to photoactivation, immediately after photoactivation (0 s), and subsequently at 15 s intervals from this movie are presented in Figure 3D.

Movie S4, Related to Figure 4. Dynamics of Photoactivated paGFP-α5 Integrin in A2780 Cells Migrating on Cell-Derived Matrix A2780-Rab25 cells were transfected with Cherry-Rab25 in conjunction with photoactivatable α5 integrin (paGFP-α5). Cells were plated onto cell-derived matrices 6 hr prior to imaging. Integrin recycling from Rab25 vesicles toward the tips of extending pseudopods was visualized using photoactivation, which was performed with a 405 nm laser aimed at Rab25-positive vesicles. Images were captured with a confocal microscope over a period of 140 s, and movies were generated from these. Stills corresponding to frames from this movie are presented in Figure 4A.

Movie S5, Related to Figure 4. Dynamics of Photoactivated paGFP-CAα5 Integrin in A2780 Cells Migrating on Cell-Derived Matrix A2780-Rab25 cells were transfected with Cherry-Rab25 in conjunction with photoactivatable constitutively active α5 integrin (paGFP-CAα5). Cells were plated onto cell-derived matrices 6 hr prior to imaging and photoactivation, and movie capture was the same as in Movie S4. Stills corresponding to frames from these movies are presented in Figure 4C.

Movie S6, Related to Figure 4. Dynamics of Photoactivated paGFP-CAα5 Integrin in A2780 Cells Migrating on Cell-Derived Matrix in the Presence of Bafilomycin A2780-Rab25 cells were transfected with Cherry-Rab25 in conjunction with photoactivatable constitutively active α5 integrin (paGFP-CAα5). Cells were plated onto cell-derived matrices as in Movie S4 and treated with bafilomycin (100 nM) 2 hr prior to imaging. Photoactivation and movie capture was the same as in Movie S4. Stills corresponding to frames from these movies are presented in Figure 4.

Movie S7, Related to Figure 5. Knockdown of CLIC3 Causes Cells to Pause When Migrating on Cell-Derived Matrices A2780-Rab25 cells were transfected with nontargeting siRNA (si-NT; see first half of movie) or with an siRNA targeting CLIC3 (si-CLIC3; see second half of movie). Transfected cells were plated onto cell-derived matrices in the presence of soluble fibronectin (2.5 μg/ml) and allowed to adhere for 24 hr prior to time-lapse microscopy. Images were captured every 5 min over an 8 hr period, and movies were generated from these. Frames from cells transfected with nontargetting siRNA (si-NT) and the CLIC3 siRNA (si-CLIC3) are presented in the first and second halves of the movie, respectively. Track plots indicating the position of the nucleus are overlayed onto the movie.
