# Supplementary material for: Rab25 and CLIC3 Collaborate to Promote Integrin Recycling from Late Endosomes/Lysosomes and Drive Cancer Progression
Source: Dev Cell. 2012 Jan 17;22(1):131–45. doi: 10.1016/j.devcel.2011.11.008 (PMC3507630; doi:10.1016/j.devcel.2011.11.008)
Supplement: Document S1. Figures S1–S5 and Table S2 [file mmc1.pdf]

Developmental Cell, Volume *22*

Supplemental Information

Rab25 and CLIC3 Collaborate to Promote Integrin Recycling from Late Endosomes/Lysosomes and Drive Cancer Progression

Marta A. Dozynkiewicz, Nigel B. Jamieson, Iain MacPherson, Joan Grindlay, Peter van den Berghe, Anne von Thun, Jennifer P. Morton, Charlie Gourley, Paul Timpson, Colin Nixon, Colin J. McKay, Ross Carter, David Strachan, Kurt Anderson, Owen J. Sansom, Patrick T. Caswell, and Jim C. Norman

SUPPLEMENTAL INVENTORY

SUPPLEMENTAL FIGURES (5 in total)

Figure S1, related to Figure 1. Validation of Rab25 and CLIC3 antibodies

Figure S2, related to Figure 2. CLIC3 and β1 integrin in A2780 cells (S2A-D. CLIC3 is a late endosomal/lysosomal protein; S2E-F, Determination of active β1 integrin in A2780 cells)

Figure S3, related to Figure 2. Single channel immunofluorescence images of merged images in Figure 2C and 2E.

Figure S4, related to Figures 3 and 4. Photoactivated paGFP-CAα5 recycles from LE/lysosomes to the plasma membrane in glass-attached cells; GFP-CAα5 moves to LAMP1 and CLIC3-positive structures in bafilomycin-treated cells plated onto CDM; Photoactivated paGFP-CAα5 recycles from CLIC3-positive vesicles to the rear of the cells plated onto CDM

Figure S5, related to Figure 4. Distribution of CLIC3, Rab25 and late endosomal-lysosomal markers in A2780-Rab25 cells migrating on CDM

SUPPLEMENTAL TABLES (2 in total)

Table S1, related to Figure 1. Microarray sample preparation, data analysis and validation and the link to the EXCEL spreadsheet which includes the full microarray dataset.

Table S2, related to Figure 7.   Predictors of survival in pancreatic cancer.

SUPPLEMENTAL MOVIES (7 in total)

Movie S1, related to Figure 2. Dynamics of GFP-Rab25 and Cherry-CLIC3 in glass-attached A2780 cells

Movie S2, related to Figure 3. Dynamics of photoactivated paGFP-α5 integrin in glass-attached A2780 cells

Movie S3, related to Figure 3. Dynamics of photoactivated paGFP-α5 integrin in glass-attached A2780 cells in the presence of bafilomycin

Movie S4, related to Figure 4. Dynamics of photoactivated paGFP-α5 integrin in A2780 cells migrating on cell-derived matrix

Movie S5, related to Figure 4. Dynamics of photoactivated paGFP-CAα5 integrin in A2780 cells migrating on cell-derived matrix

Movie S6, related to Figure 4. Dynamics of photoactivated paGFP-CAα5 integrin in A2780 cells migrating on cell-derived matrix in the presence of bafilomycin

Movie S7, related to Figure 5. Knockdown of CLIC3 causes cells to pause when migrating on cell-derived matrices

SUPPLEMENTAL FIGURES

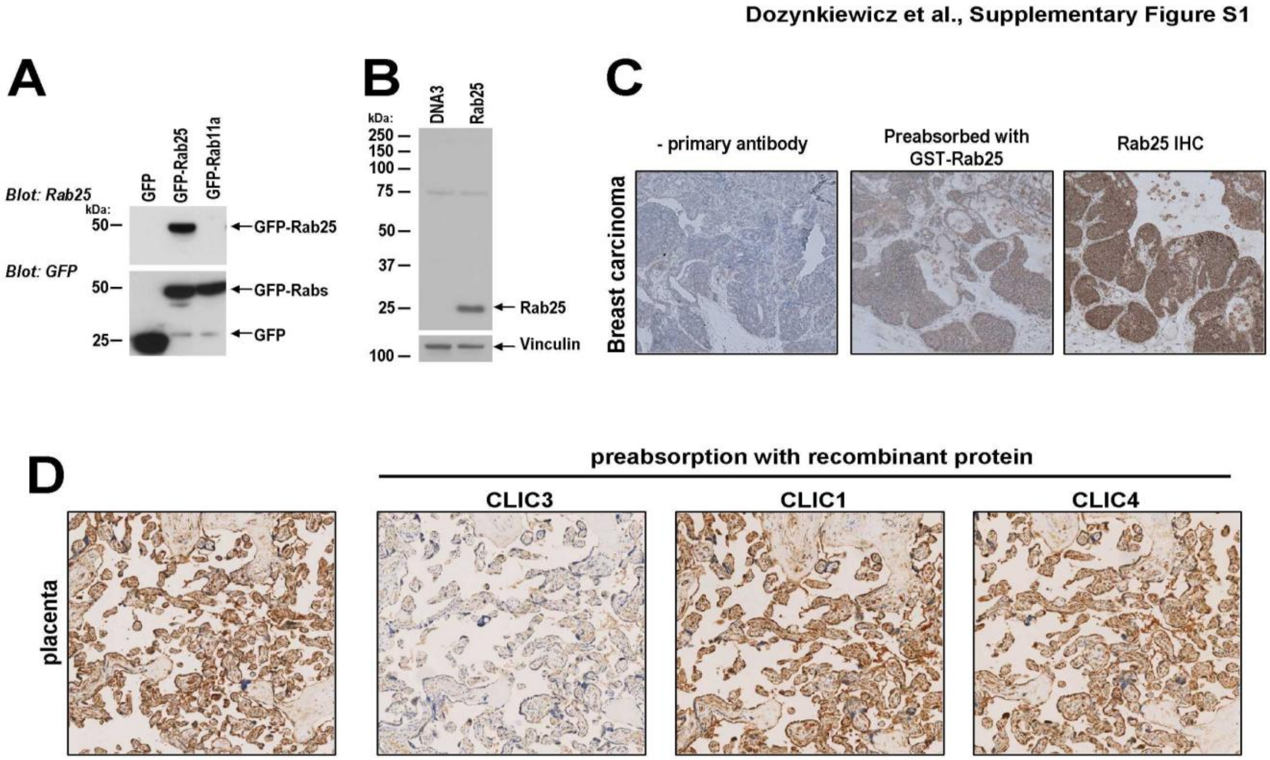

Figure S1, related to Figure 1. Validation of Rab25 and CLIC3 antibodies

(A) Western blot of A2780 cells transfected with GFP, GFP-Rab25 or GFP-Rab11a showing specificity of the antibody towards Rab25. **(B)** Rab25 antibody recognises a single band in A2780-Rab25 cells lysates, but not in A2780-DNA3 lysates. **(C)** Rab25 antibody controls for immunohistochemical staining performed on human breast carcinoma in the absence of primary antibody, in the presence of the antibody and following a preabsorption control. **(D)** Immunohistochemical analysis of CLIC3 in human placenta (a CLIC3-rich tissue); the preabsorption controls indicate specificity of the antibody for CLIC3 over CLICs 1 and 4.

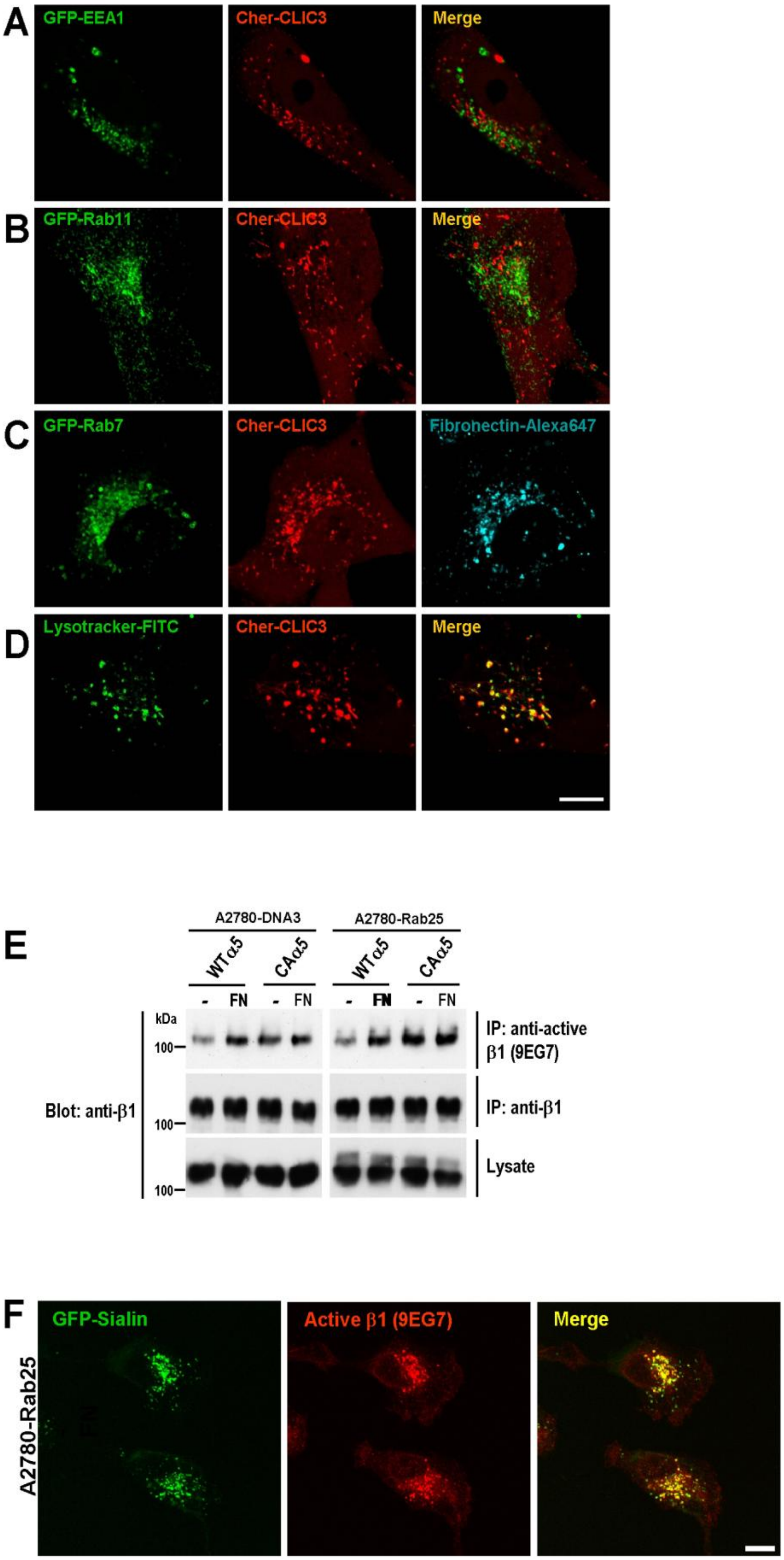
A
GFP-EEA1
Cher-CLIC3
Merge
B
GFP-Rab11
Cher-CLIC3
Merge
C
GFP-Rab7
Cher-CLIC3
Fibronectin-Alexa647
D
Lysotracker-FITC
Cher-CLIC3
Merge
E
A2780-DNA3
A2780-Rab25
WTα5
CAα5
WTα5
CAα5
- FN - FN - FN - FN
kDa
100
IP: anti-active β1 (9EG7)
Blot: anti-β1
100
IP: anti-β1
100
Lysate
F
GFP-Sialin
Active β1 (9EG7)
Merge
A2780-Rab25

Figure S2, related to Figure 2. CLIC3 and β1 integrin in A2780 cells

S2A-C. CLIC3 is a late endosomal-lysosomal protein. A2780-Rab25 cells were transfected with Cherry-CLIC3 (red) and GFP-EEA1 (A), GFP-Rab11 (B), GFP-Rab7 (C) or Cherry-CLIC3 alone (D). Cells in (C) were incubated with 2.5 μg/ml soluble fibronectin-Alexa647 and in (D) with lysotracker-FITC (100nM). The distribution of the fluorescent proteins and probes was determined by live cell confocal microscopy. Bars, 10μm. **S2E-F. Determination of active β1 integrin in A2780 cells. (E)** A2780-DNA3 and A2780-Rab25 cells were transfected with GFP-α5 or GFP-CAα5, and then incubated in the presence or absence of fibronectin (FN; 3.0 μg/ml) for 3 hr. Cells were lysed and total (anti-β1) and active (9EG7) β1 integrins were immunoprecipitated. The amount of β1 integrin present in the immunoprecipitates and in the lysates was determined by Western blotting. **(F)** A2780-Rab25 cells were incubated with fibronectin (FN; 3.0 μg/ml) for 3 hr and active β1 integrin (9EG7) visualised with respect to a marker of late endosomes-lysosomes (sialin) by confocal microscopy. Bar, 10μm. The focal plane is across the centre of the nucleus.

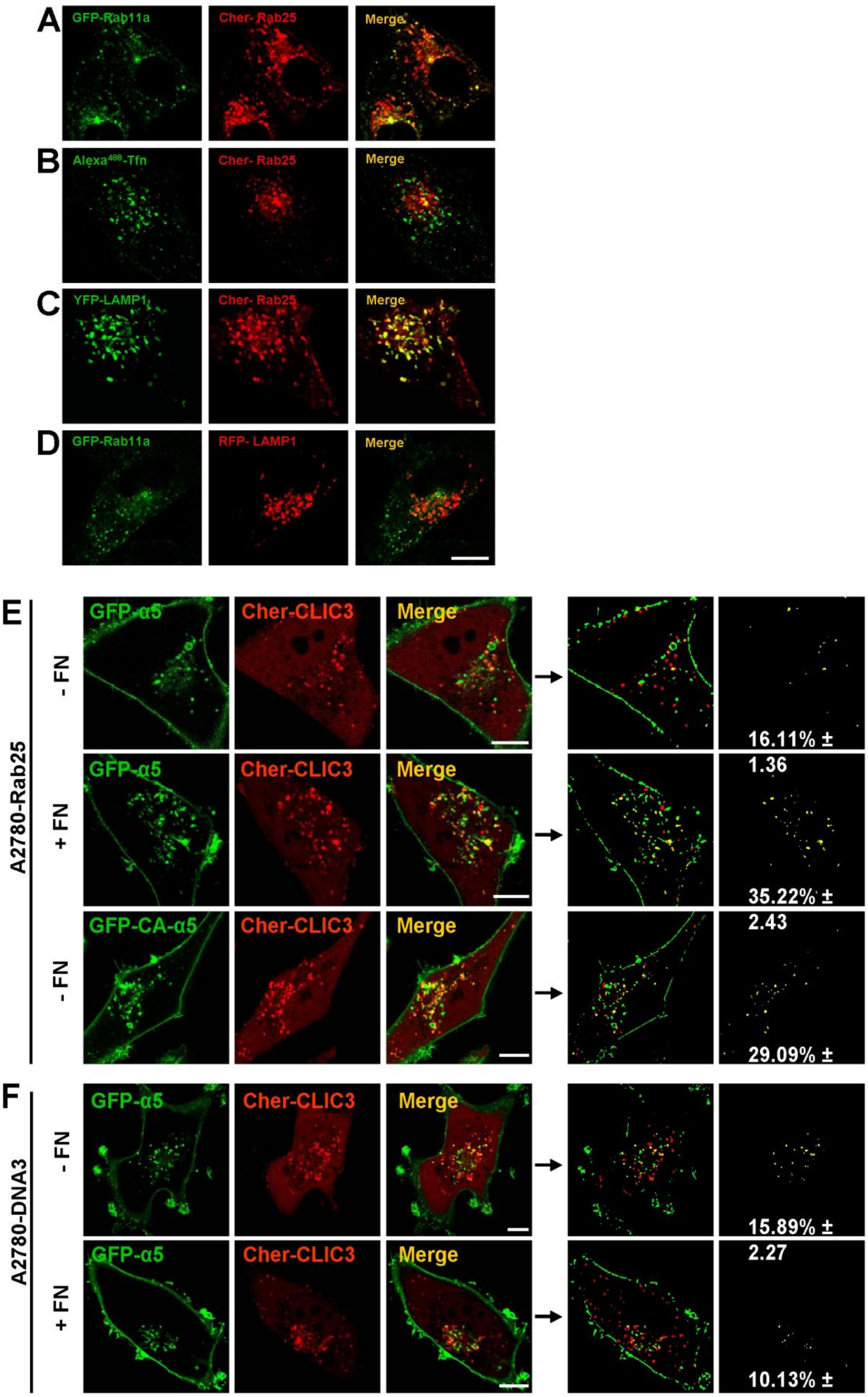
A
GFP-Rab11a
Cher- Rab25
Merge
B
Alexa488-Tfn
Cher- Rab25
Merge
C
YFP-LAMP1
Cher- Rab25
Merge
D
GFP-Rab11a
RFP- LAMP1
Merge
E
A2780-Rab25
- FN
GFP-α5
Cher-CLIC3
Merge
16.11% ±
1.36
+ FN
GFP-α5
Cher-CLIC3
Merge
35.22% ±
2.43
- FN
GFP-CA-α5
Cher-CLIC3
Merge
29.09% ±
F
A2780-DNA3
- FN
GFP-α5
Cher-CLIC3
Merge
15.89% ±
2.27
+ FN
GFP-α5
Cher-CLIC3
Merge
10.13% ±

Figure S3, related to Figure 2. Single channel immunofluorescence images of merged images in Figure 2C and 2E.

A-D. Localisation of Rab25 with respect to markers of late and recycling endosomes. A2780-Rab25 cells were transfected with Cherry-Rab25 (A-C) or RFP-LAMP1 (D) in combination with GFP-Rab11A (A, D) or YFP-LAMP1 (C). In (B) the cells were incubated with Alexa^{488}-transferrin (10μg/ml) for 30 min. (B). The distribution of the fluorescent proteins was determined by live confocal microscopy, and stills from these movies are displayed. Bar, 10 μm. These images represent the individual green and red fluorescence channel images from Fig. 2C. **E-F. Rab25 is required for sorting of active α5β1 to CLIC3 positive late endosomes-lysosomes**. A2780-Rab25 (E) or A2780-DNA3 (F) cells were transfected with Cherry-CLIC3 (red) and either α5 integrin (GFP-α5) or a constitutively active mutant of this protein (GFP-CA-α5) and incubated with or without fibronectin (FN, 2.5μg/ml). Live cell confocal imaging was used to visualise the localisation of the fluorescent proteins. Image J was used to quantify colocalisation between CLIC3 and α5 (depicted in the right hand side panel of contrast enhanced images), which is expressed as the percentage of yellow pixels versus red pixels. Data are mean ± SEM from at least 4 experiments, n>30 cells. Bars, 10μm. Merged images and quantification is also shown in figure 2E.

Dozynkiewicz et al., Supplementary Figure S4

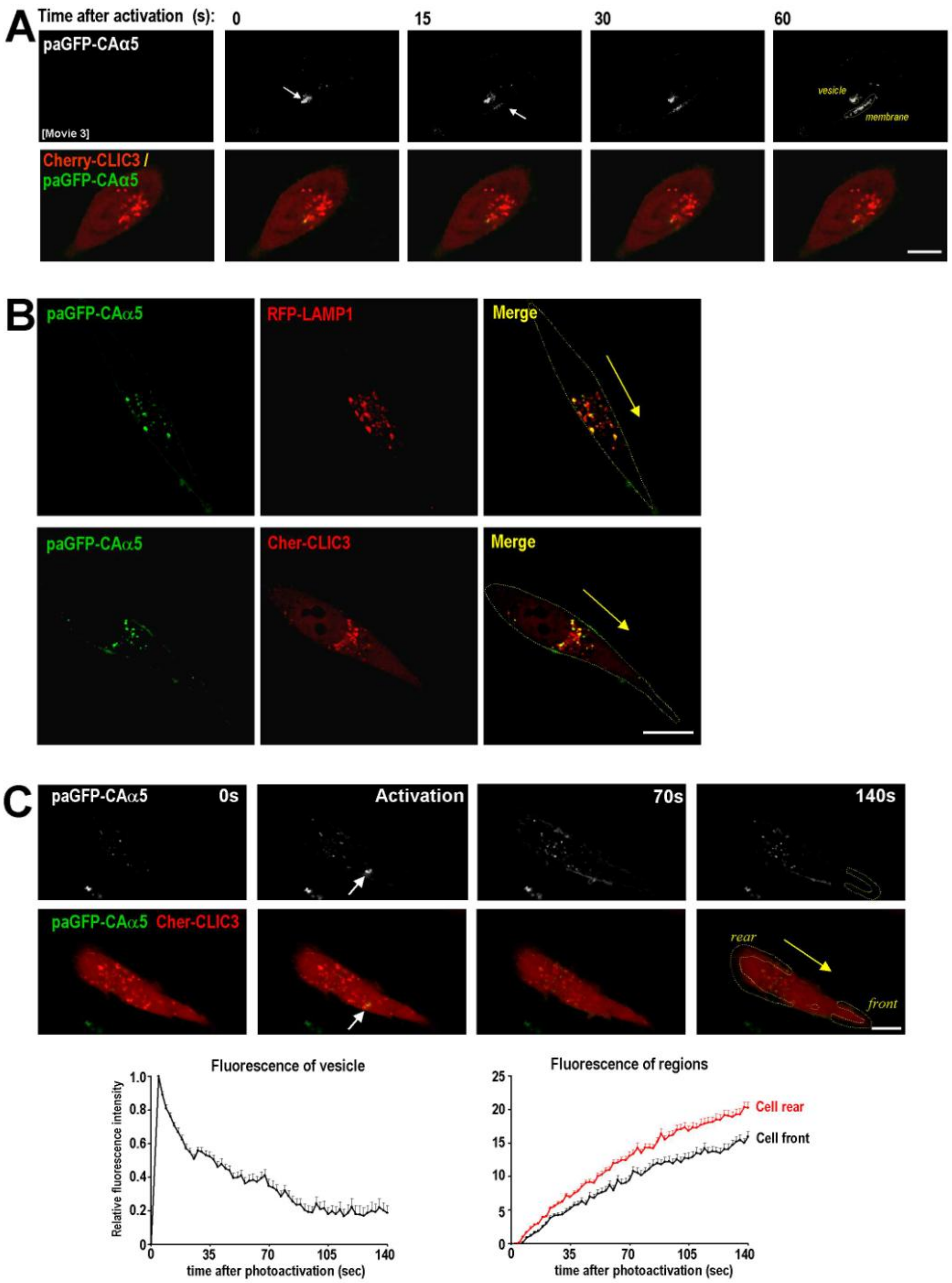

Figure S4, related to Figure 3 and 4.

(A) Photoactivated paGFP-CAα5 recycles from late endosomes-lysosomes to the plasma membrane in glass-attached cells

A2780-Rab25 cells were transfected with Cherry-CLIC3 in conjunction with photoactivatable constitutively active α5 integrin (paGFP-CAα5). Photoactivation was performed with a 405nm laser aimed at a point corresponding to a CLIC3-positive vesicle (arrow). Images were captured and movies generated as for Fig 3. Bar, 10µm. Quantification of movies corresponding to this representative one is shown in Fig 3E.

(B) GFP-CAα5 moves to LAMP1 and CLIC3-positive structures in bafilomycin-treated cells plated onto CDM

A2780-Rab25 cells were transfected with Cherry-CLIC3 or RFP-LAMP1 in conjunction with constitutively active α5 integrin (GFP-CAα5) and plated onto CDM. Cells were treated with bafilomycin (100nM) for 2hr and images captured with a confocal microscope. Bar, 10µm. Yellow dotted line describes the cell periphery, the arrow the direction of migration.

(C) Photoactivated paGFP-CAα5 recycles from CLIC3-positive vesicles to the rear of cells plated onto CDM

A2780-Rab25 cells were transfected with Cherry-CLIC3 in conjunction with photoactivatable constitutively active α5 integrin (paGFP-CAα5). Cells were plated onto CDM 6 hours prior to imaging and integrin recycling was visualised using photoactivation, which was performed with a 405nm laser aimed at CLIC3-positive vesicles, as denoted by the white arrows. Images were captured and movies generated as for Fig 4. Bars, 10µm. Yellow arrow indicates the direction of migration. The integrated fluorescence intensity of the photoactivated region and plasma membrane regions at the cell front and cell rear (yellow dotted lines) was calculated for each frame of the movie. Relative fluorescence intensity (vesicle) or percentage increase in fluorescence intensity (region) was plotted against time.

Dozynkiewicz et al., Supplementary Figure S5

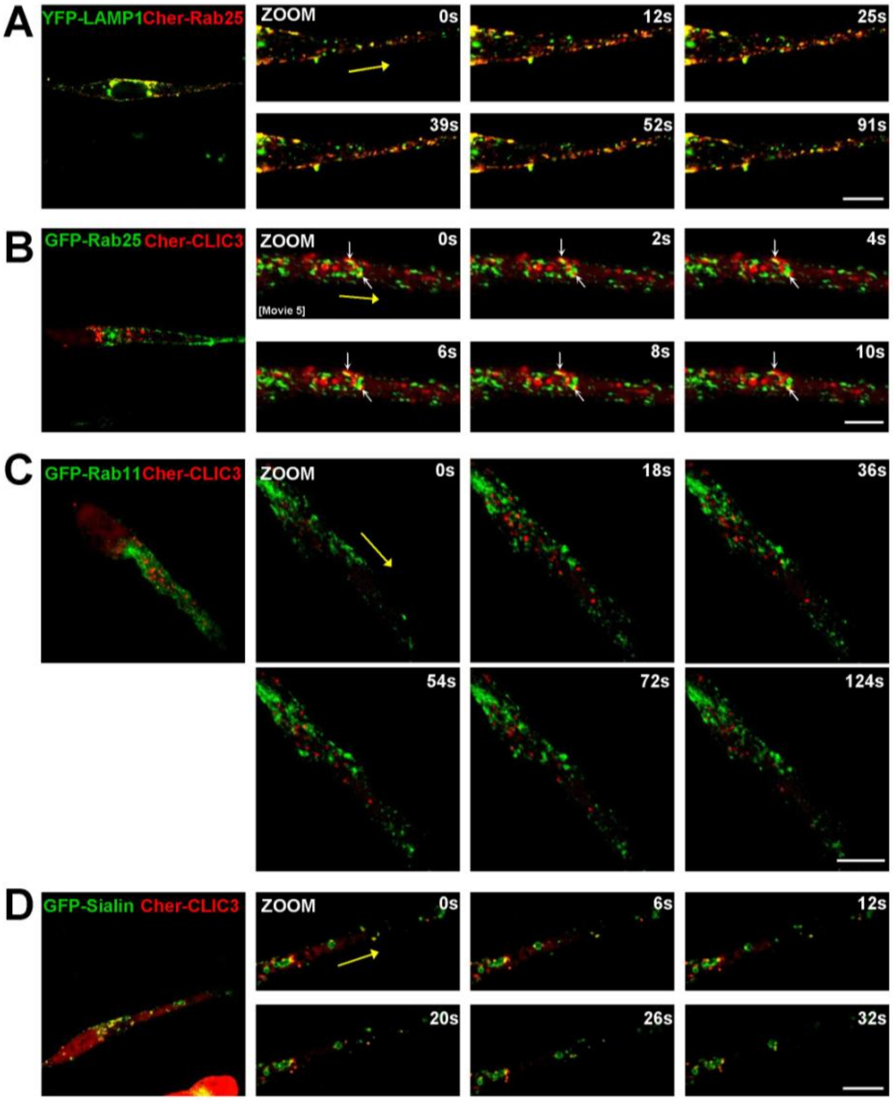

Figure S5, related to Figure 4. Distribution of CLIC3, Rab25 and late endosomal/lysosomal markers in A2780-Rab25 cells migrating on CDM

A2780-Rab25 cells were transfected with Cherry-CLIC3 (red) in conjunction with YFP-LAMP1 (A), GFP-Rab25 (B), GFP-Rab11 (C), or GFP-sialin (D) and plated onto dishes coated with CDM. Cells were incubated with 2.5 μg/ml soluble fibronectin prior to imaging and the distribution of fluorescent proteins was visualised by live cell confocal microscopy. Movies were captured and 4 zoomed and merged stills from these are shown, together with the whole-cell view (left hand side). Yellow arrow indicates the direction of migration. Bars, 10μm.

SUPPLEMENTAL TABLES

Table S1, related to Figure 1. Microarray sample preparation, data analysis and validation

(See accompanying Excel file)

Cells were plated onto plastic and CDMs for 16 hours and RNA was extracted using RNeasy kit (Qiagen). Biotinylated cRNA was generated and hybridised to GeneChip® human genome U133 Plus 2.0 chip by Cancer Research UK Paterson Institute Microarray Service. The Cel files were normalized and analyzed in Partek® Genomics Suite Software (version 6.5beta Copyright © 2009). Default GCRMA normalization and log2 transformation of the data was followed by the removal of technical batch effects. Multiway ANOVA was used to identify significantly regulated genes from one of experimental groups and linear contrasts performed between all pairs of experimental conditions. Multiple test corrections were performed for all calculated p-values. Less conservative step up p-value, a p-value that controls the false discovery rate was used for further ranking of significantly differentially expressed genes. Finally, the fold change values were considered in ranking genes of interest and these data are presented in the supplementary table/EXCEL spreadsheet S1.

Table S2, related to Figure 7. Predictors of survival in PDAC

(A) Predictors of survival in 118 patients with PDAC following pancreaticoduodenectomy according to multivariate Cox Regression analysis. CLIC3 protein expression was assessed by immunohistochemistry.

		**Overall survival**	
		HR (95% CI)	P value
Age (years)	<65/≥65	0.83 (0.67-1.03)	0.099
Gender	Female/Male	1.01 (0.62-1.61)	0.991
Tumour stage	T2/T3	2,20 (1.13-4.27)	0.019
Tumour size (mm)	<30/≥30	1.82 (1.19-2.79)	0.006
Lymph node status	Absent/Present	1.71 (0.92-3.23)	0.091
Margin involvement	R0/R1	2.23 (1.32-3.77)	0.003
Tumour grade	Low/High	1.98 (1.25-3.14)	0.004
Perineural invasion	Absent/Present	0.73 (0.27-1.92)	0.519
Vascular invasion	Absent/Present	1.32 (0.89-2.09)	0.213
CLIC3	Low/High	1.35 (1.08-1.81)	0.028

(B) Predictors of survival in 48 patients with PDAC following pancreaticoduodenectomy according to multivariate Cox Regression analysis. CLIC3 mRNA expression was assessed by microarray hybridisation.

		**Overall survival**	
		HR (95% CI)	P value
Tumour stage	T2/T3	2.29 (1.35-3.82)	0.023
Tumour size (mm)	<30/≥30	2.67 (1.08-6.57)	0.033
Lymph node status	Absent/Present	2.43 (0.81-9.11)	0.199
Margin involvement	R0/R1	2.83 (1.77-4.14)	0.002
Tumour grade	Low/High	1.83 (0.71-4.68)	0.21
Vascular invasion	Absent/Present	2.10 (0.78-5.68)	0.143
CLIC3	Low/High	4.81 (1.86-12.3)	0.001
