# Supplementary figures and images for: Rab25 and CLIC3 Collaborate to Promote Integrin Recycling from Late Endosomes/Lysosomes and Drive Cancer Progression
Source: Dev Cell. 2012 Jan 17;22(1):131–45. doi: 10.1016/j.devcel.2011.11.008 (PMC3507630; doi:10.1016/j.devcel.2011.11.008)

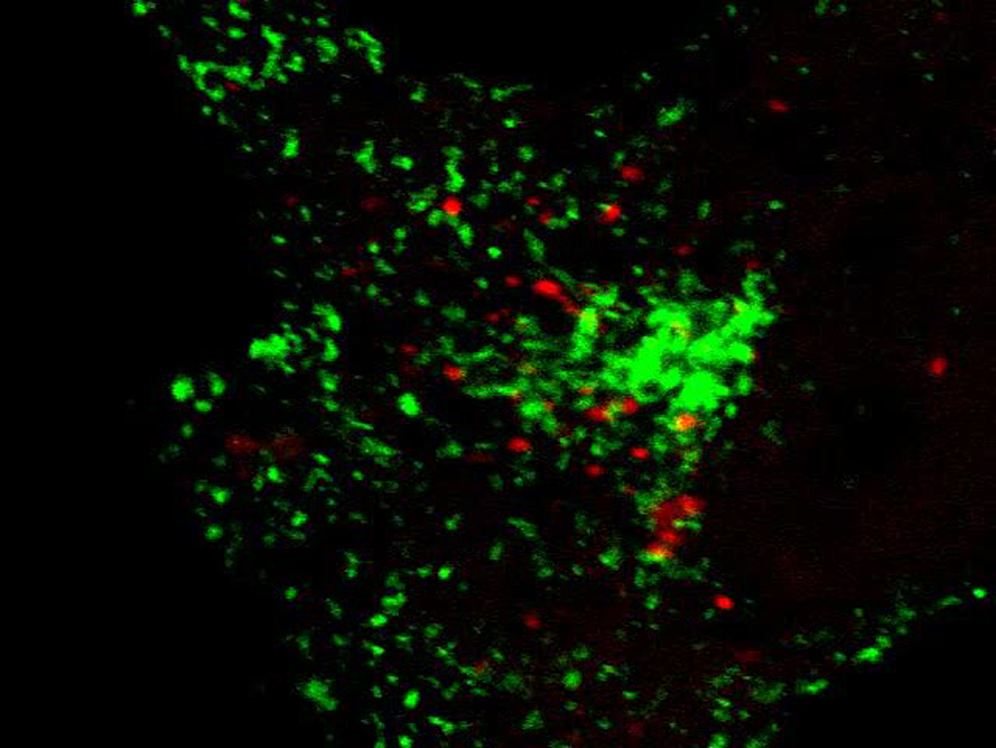

Supplement: Movie S1, Related to Figure 2. Dynamics of GFP-Rab25 and Cherry-CLIC3 in Glass-Attached A2780 Cells — A2780-Rab25 cells were transfected with GFP-Rab25 in combination with Cherry-CLIC3 and plated onto glass dishes. The distribution of the fluorescent proteins was determined by live confocal microscopy with images being captured at 2 s intervals over a period of 2 min. The stills in Figure 2D are extracted from this movie. [file mmc3.jpg]

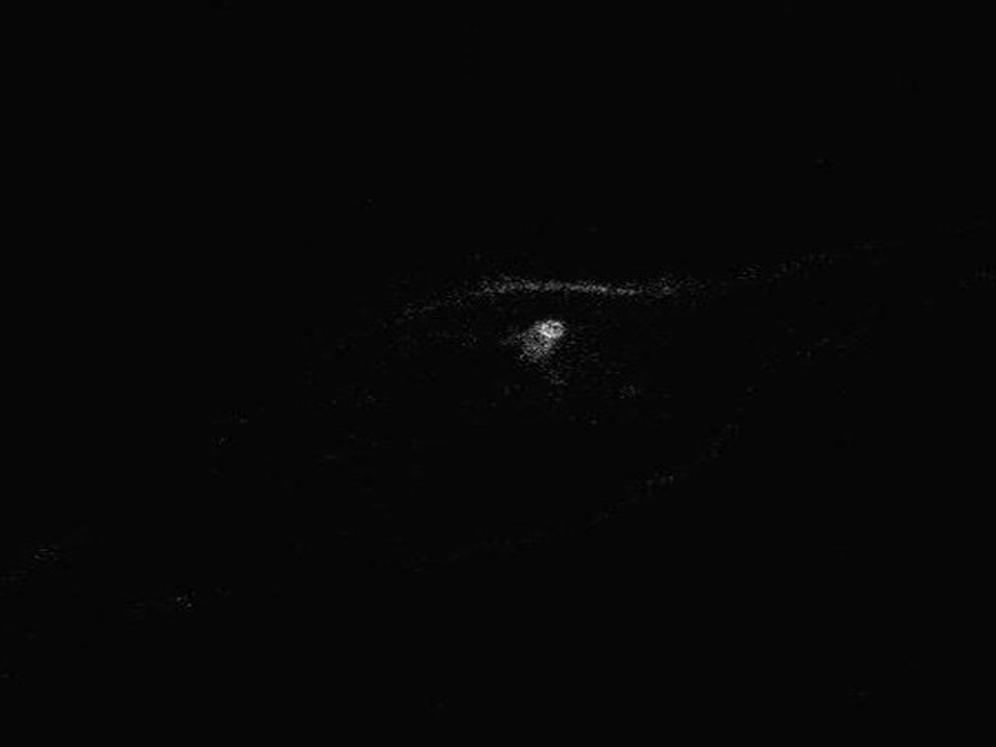

Supplement: Movie S2, Related to Figure 3. Dynamics of Photoactivated paGFP-α5 Integrin in Glass-Attached A2780 Cells — A2780-Rab25 cells were transfected with Cherry-CLIC3 in conjunction with photoactivatable α5 integrin (paGFP-α5). Cells were incubated with fibronectin (FN; 2.5 μg/ml). Integrin recycling from CLIC3 vesicles was visualized using photoactivation, which was performed with a 405 nm laser aimed at CLIC3-positive vesicles. Images were captured with a confocal microscope every 2 s over a period of 120 s. Stills corresponding to frames prior to photoactivation, immediately after photoactivation (0 s), and subsequently at 15 s intervals from this movie are presented in Figure 3D. [file mmc4.jpg]

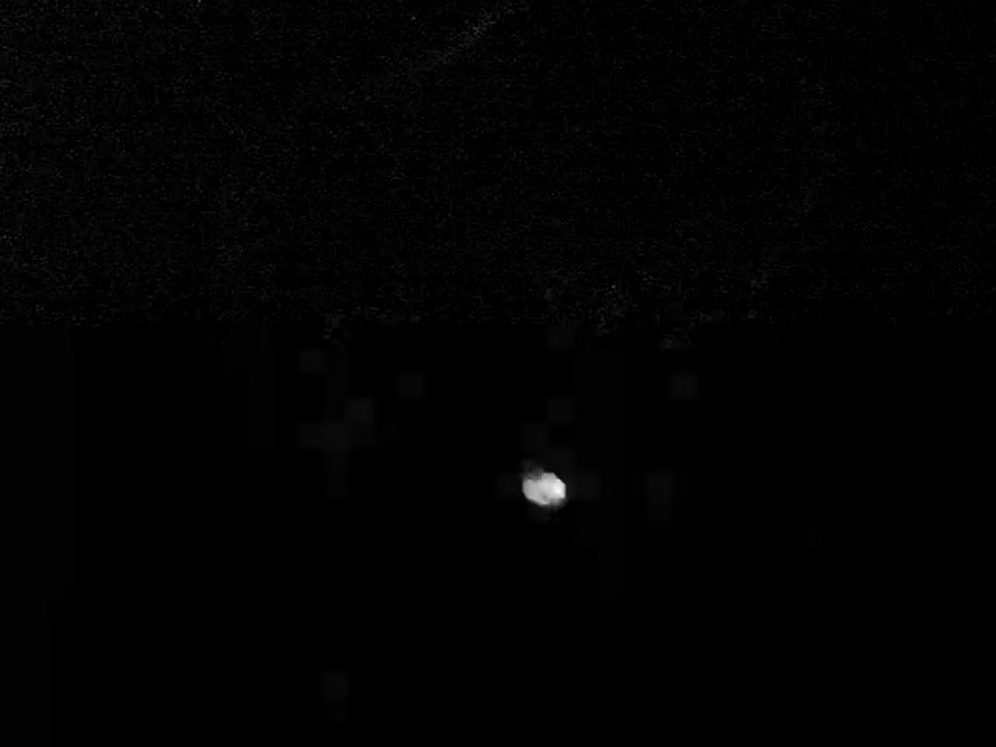

Supplement: Movie S3, Related to Figure 3. Dynamics of Photoactivated paGFP-α5 Integrin in Glass-Attached A2780 Cells in the Presence of Bafilomycin — A2780-Rab25 cells were transfected with Cherry-CLIC3 in conjunction with photoactivatable α5 integrin (paGFP-α5). Cells were incubated with fibronectin (FN; 2.5 μg/ml) in conjunction with bafilomycin (BAF; 100 nM). Integrin recycling from CLIC3 vesicles was visualized using photoactivation, which was performed with a 405 nm laser aimed at CLIC3-positive vesicles. Images were captured with a confocal microscope every 2 s over a period of 120 s. Stills corresponding to frames prior to photoactivation, immediately after photoactivation (0 s), and subsequently at 15 s intervals from this movie are presented in Figure 3D. [file mmc5.jpg]

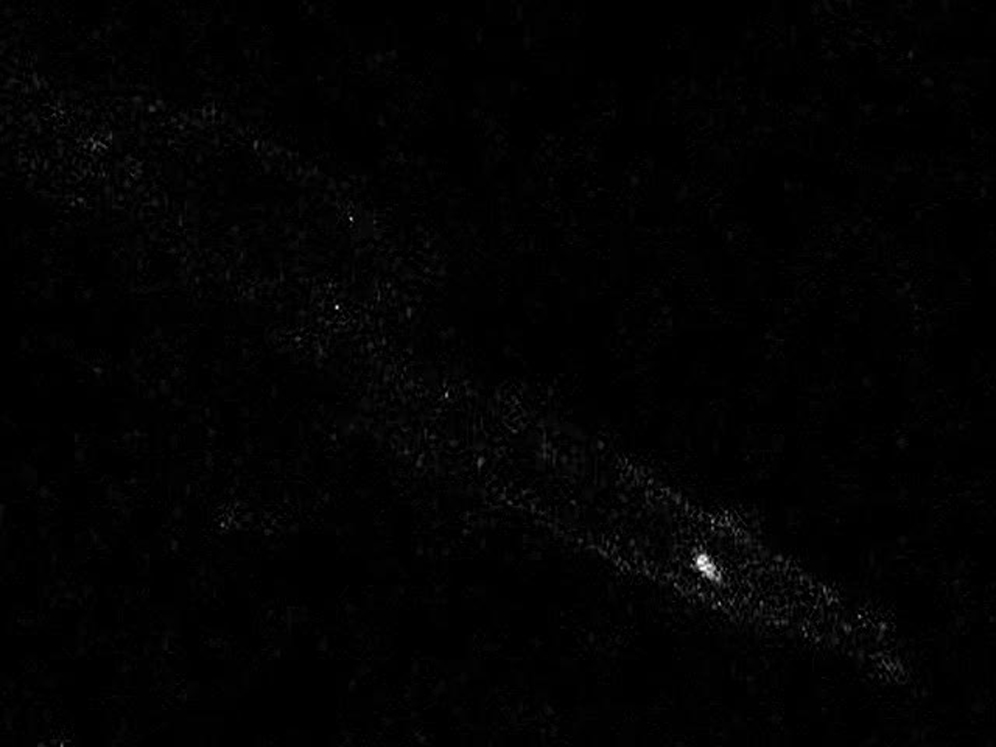

Supplement: Movie S4, Related to Figure 4. Dynamics of Photoactivated paGFP-α5 Integrin in A2780 Cells Migrating on Cell-Derived Matrix — A2780-Rab25 cells were transfected with Cherry-Rab25 in conjunction with photoactivatable α5 integrin (paGFP-α5). Cells were plated onto cell-derived matrices 6 hr prior to imaging. Integrin recycling from Rab25 vesicles toward the tips of extending pseudopods was visualized using photoactivation, which was performed with a 405 nm laser aimed at Rab25-positive vesicles. Images were captured with a confocal microscope over a period of 140 s, and movies were generated from these. Stills corresponding to frames from this movie are presented in Figure 4A. [file mmc6.jpg]

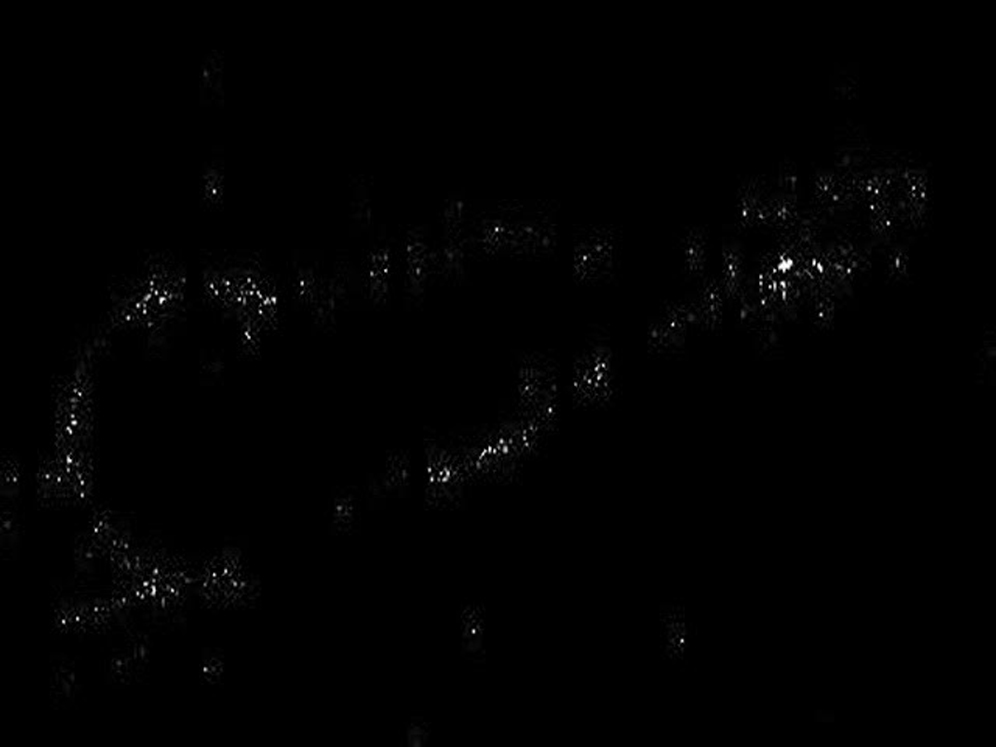

Supplement: Movie S5, Related to Figure 4. Dynamics of Photoactivated paGFP-CAα5 Integrin in A2780 Cells Migrating on Cell-Derived Matrix — A2780-Rab25 cells were transfected with Cherry-Rab25 in conjunction with photoactivatable constitutively active α5 integrin (paGFP-CAα5). Cells were plated onto cell-derived matrices 6 hr prior to imaging and photoactivation, and movie capture was the same as in Movie S4. Stills corresponding to frames from these movies are presented in Figure 4C. [file mmc7.jpg]

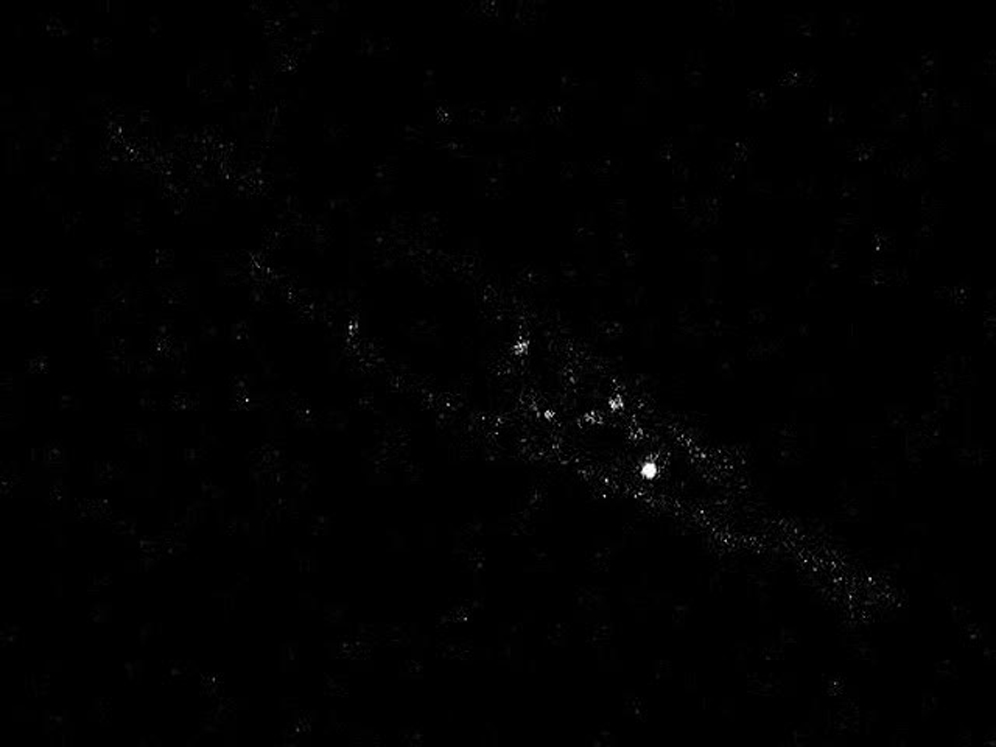

Supplement: Movie S6, Related to Figure 4. Dynamics of Photoactivated paGFP-CAα5 Integrin in A2780 Cells Migrating on Cell-Derived Matrix in the Presence of Bafilomycin — A2780-Rab25 cells were transfected with Cherry-Rab25 in conjunction with photoactivatable constitutively active α5 integrin (paGFP-CAα5). Cells were plated onto cell-derived matrices as in Movie S4 and treated with bafilomycin (100 nM) 2 hr prior to imaging. Photoactivation and movie capture was the same as in Movie S4. Stills corresponding to frames from these movies are presented in Figure 4. [file mmc8.jpg]

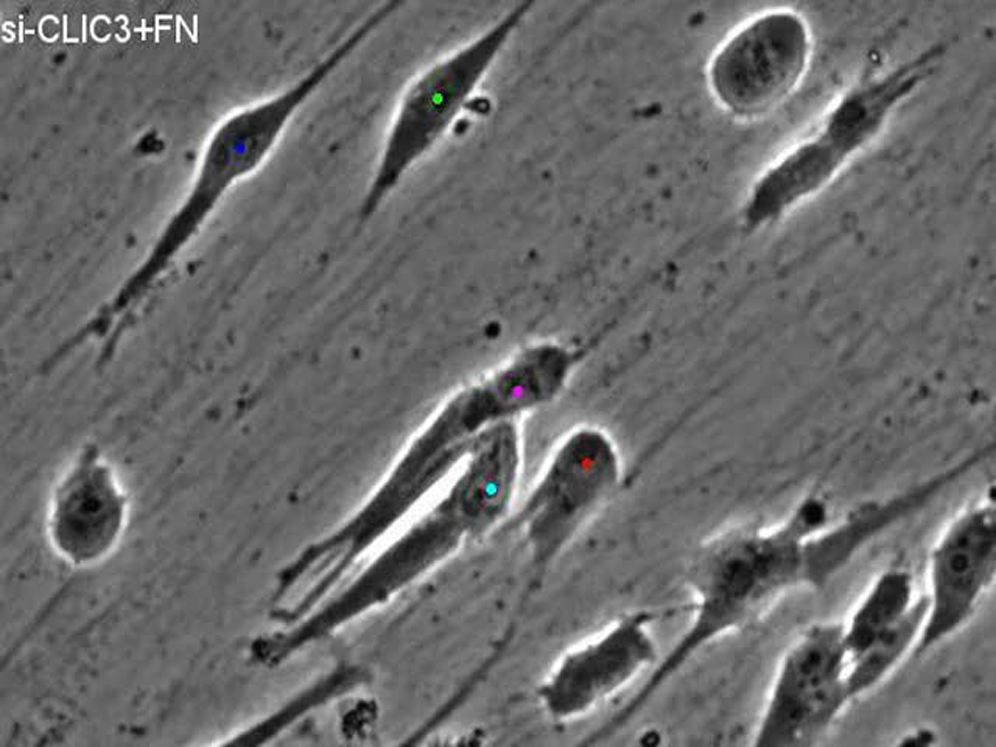

Supplement: Movie S7, Related to Figure 5. Knockdown of CLIC3 Causes Cells to Pause When Migrating on Cell-Derived Matrices — A2780-Rab25 cells were transfected with nontargeting siRNA (si-NT; see first half of movie) or with an siRNA targeting CLIC3 (si-CLIC3; see second half of movie). Transfected cells were plated onto cell-derived matrices in the presence of soluble fibronectin (2.5 μg/ml) and allowed to adhere for 24 hr prior to time-lapse microscopy. Images were captured every 5 min over an 8 hr period, and movies were generated from these. Frames from cells transfected with nontargetting siRNA (si-NT) and the CLIC3 siRNA (si-CLIC3) are presented in the first and second halves of the movie, respectively. Track plots indicating the position of the nucleus are overlayed onto the movie. [file mmc9.jpg]
